# Multi-Modal Design, Synthesis, and Biological Evaluation of Novel Fusidic Acid Derivatives

**DOI:** 10.3390/molecules30091983

**Published:** 2025-04-29

**Authors:** Luqi Wang, Zhiyuan Geng, Yuhang Liu, Linhui Cao, Yao Liu, Hourui Zhang, Yi Bi, Jing Lu

**Affiliations:** School of Pharmacy, Key Laboratory of Molecular Pharmacology and Drug Evaluation (Yantai University), Ministry of Education, Collaborative Innovation Center of Advanced Drug Delivery System and Biotech Drugs in Universities of Shandong, Yantai University, Yantai 264005, China; phawanglq@163.com (L.W.); gzy15224366434@163.com (Z.G.); lyh182243667592023@163.com (Y.L.); clh9785@126.com (L.C.); lyliuyao2002@163.com (Y.L.); a15063257073@163.com (H.Z.)

**Keywords:** fusidic acid derivative, molecular dynamics simulation, AI-based drug design and screening, in vitro model

## Abstract

Fusidic acid (FA), a tetracyclic triterpenoid, has been approved to treat methicillin-resistant Staphylococcus aureus (MRSA) infections. However, there are few reports about FA derivatives with high efficacy superior to FA, manifesting the difficulty of discovering the derivatives based on experience-based drug design. In this study, we employed a stepwise method to discover novel FA derivatives. First, molecular dynamics (MD) simulations were performed to identify the molecular mechanism of FA against elongation factor G (EF-G) and drug resistance. Then, we utilized a scaffold decorator to design novel FA derivatives at the 3- and 21-positions of FA. The ligand-based and structure-based screening models, including Chemprop and RTMScore, were employed to identify promising hits from the generated set. Ten generated FA derivatives with high efficacy in the Chemprop and RTMScore models were synthesized for in vitro testing. Compounds **4** and **10** demonstrated a 2-fold increase in potency against MRSA strains compared to FA. This study highlights the significant impact of AI-based methods on the design of novel FA derivatives with drug efficacy, which provides a new approach for drug discovery.

## 1. Introduction

Staphylococcus aureus (*S. aureus*) is a major Gram-positive bacterial pathogen in community-acquired and hospital-acquired infections, causing diseases ranging from skin and soft tissue infections to life-threatening conditions such as severe pneumonia and septic shock [1,2]. Methicillin-resistant Staphylococcus aureus (MRSA) infection is the most common type of *S. aureus* infection, characterized by a higher mortality rate compared to infections caused by non-resistant bacteria due to the antibiotic resistance of MRSA against commonly used antibiotics for these infections [3]. Therefore, MRSA was listed as a high-priority antibiotic-resistant priority pathogen by the World Health Organization [4]. First-line treatments for MRSA infections, such as vancomycin and linezolid, demonstrate high efficacy against complicated skin and soft tissue infections [5,6,7,8], but they also incur nephrotoxicity [9,10,11]. Developing new drugs against MRSA infections to improve their clinical therapeutic profiles is thus urgently needed.

**Figure 1:**
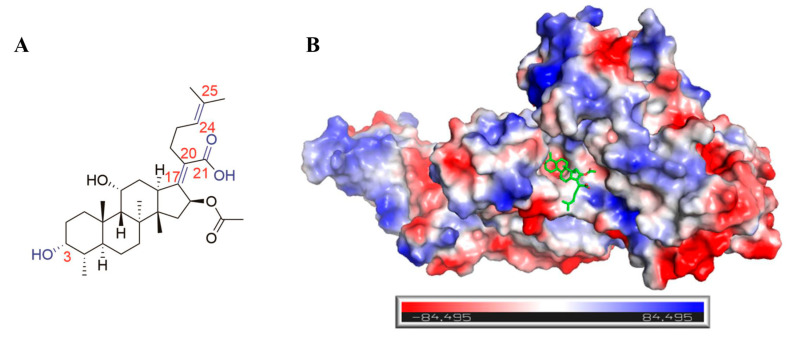
Structure of fusidic acid (FA, (**A**)) and its binding mode with EF-G (**B**). (**A**) The red label indicates the atomic numbering of FA. (**B**) FA is displayed as the green stick, and the protein surface is colored according to its electrostatic potential from red (−84.495) to blue (84.495).

Fusidic acid (FA, Figure 1A) is a tetracyclic triterpenoid isolated from fungi [12] and has been clinically used to treat systemic and localized MRSA infections [13,14]. Jones et al. reported that among 15 antimicrobial agents against *S. aureus*, including vancomycin and linezolid, FA exhibited the lowest inhibitory concentration with MIC_50_ and MIC_90_ values of 0.12 and 0.25 μg/mL, respectively [15]. FA shows a unique antibacterial mechanism of action by locking elongation factor G (EF-G) in an intermediate conformation between GTP-bound and GDP-bound states after GTP hydrolysis and translocation (Figure 1B), thereby preventing the release of the EF-G∙GDP complex in the post-translocation state and obstructing protein synthesis [16]. FA exerts efficacy against MRSA through this specific mechanism while avoiding cross-resistance to other antimicrobials, including antibiotics [17,18].

A series of FA derivatives has previously been disclosed, but only a few compounds have been obtained with equivalent potency compared to FA. For example, introducing a spiro-cyclopropane group at the δ-17(20) double bond or 17(S), 20(S)-dihydro derivatives exhibited identical activity to FA against *S. aureus* [19,20]. The derivative obtained by substituting the 16-position with 16β-acetylthio (IC_50_ = 0.013 μg/mL) exhibited slightly higher activity against *S. aureus* compared to FA (IC_50_ = 0.025 μg/mL) [21]. We designed a long-acting FA derivative by introducing chlorine-substituted benzotriazole on the 21-position in FA [22]. In addition, we and other groups have also found that FA derivatives, modified by substituting at the 3-, 21-, and 24(25)-positions, exhibit efficacy against malaria, inflammation, and cancer [23,24,25]. However, few researchers have discovered novel FA derivatives that are highly potent against MRSA [26].

**Figure 2:**
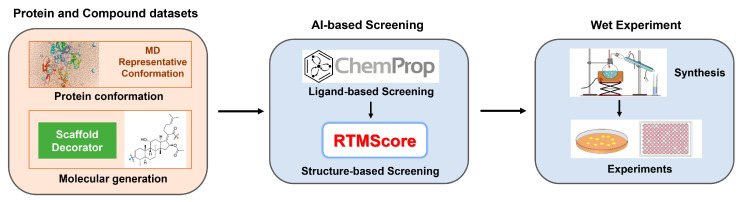
The architecture of the de novo design for novel FA derivatives, based on stepwise AI-driven drug discovery and design, and wet experiments.

The difficulty in discovering novel FA derivatives underscores the importance of innovative drug design methods in achieving superior efficacy. To address this challenge, we started by analyzing the binding-related features of FA in wild and mutant EF-Gs by molecular dynamics simulation (Figure 2). We then constructed a novel dataset of FA derivatives using a scaffold decorator [23] and further evaluated the potency of FA derivatives through ligand- and structure-based virtual screening [27,28]. The potencies of these novel compounds were explored in a translational approach: We synthesized 10 promising compounds concerning superior scores. We evaluated their inhibition rate and MIC values against MRSA. The resulting hits, **4** and **10**, exhibited the better antibacterial activity against MRSA, superior to FA.

## 2. Results and Discussion

### 2.1. Structural Basis of EF-G∙FA

**Figure 3:**
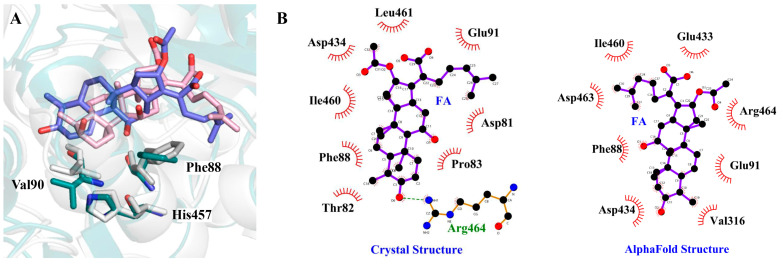
Structural basis of FA bound to EF-G. (**A**) Structural superimposition of the EF-G∙FA binding from Cryo-EM structures (PDB ID: 8P2H with white; FA with pink) and docking using SYBYL-X 2.1.1 (EF-G with forest; FA with blue). (**B**) The 2D plot of EF-G∙FA interactions from the Cryo-EM and docking modes.

At the outset of this work, the complex of EF-G and FA had not been published; therefore, we downloaded the prediction structure of EF-G from AlphaFold DB [29,30], and the modeling of FA was carried out using molecular docking to identify the binding mode. In June 2024, a Cryo-EM structure of the EF-G·FA complex was published (PDB ID: 8P2H). Our comparison analysis revealed that the AlphaFold-predicted EF-G structure shared a similar binding style with the published Cryo-EM structure, with an RMSD of 1.691 Å (Figure 3A). When focusing on the region within 5 Å of the ligand, the RMSD decreased to 0.612 Å, indicating high consistency of the functional region for ligand recognition between the two structures. The docking mode of FA in EF-G, as predicted by AlphaFold, captured key interactions (Figure 3B), providing a visual representation of the accuracy of the initial homology model. The side chain at the C20-position in FA was inserted in a hydrophobic region formed by F88, L456, and I460 and formed a hydrophobic interaction with the key residue F88. However, the hydroxyl oxygen of FA lost a hydrogen bond with the guanidinium nitrogen of Arg464 in homology EF-G due to the position of Arg464 in the homology structure being reversed compared to that in the Cryo-EM structure.

### 2.2. Comparison Between Wildtype and Mutant EF-Gs

**Figure 4:**
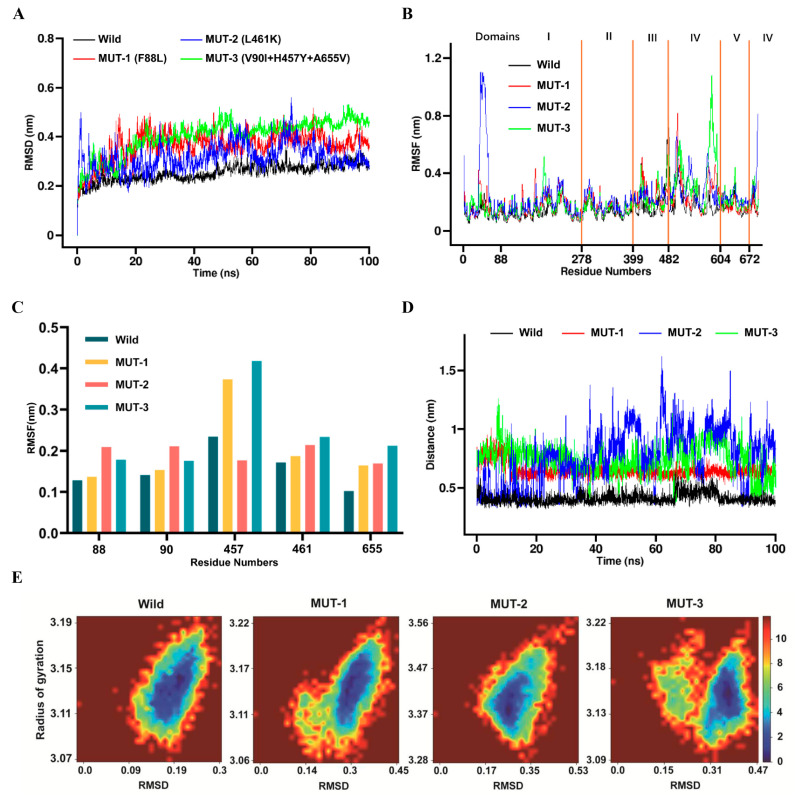
MD simulations of FA and wildtype or mutant EF-Gs. (**A**) The RMSD values of Wild (black), MUT-1 (F88L; red), MUT-2 (L461K; blue), and MUT-3 (V90I+H457Y+A655V; green) were calculated from the backbone after least squares fitting to the backbone. (**B**) The RMSF values are calculated according to the protein. The protein is divided into domains I (residues 1–277), II (residues 278–398), III (residues 399–481), IV (residues 482–603 and 672–693), and V (residues 604–671) by the orange lines. (**C**) The RMSF values of the key residues from the different types of EF-G. (**D**) The distances were measured between the α-carbon (Cα) of Phe88 (or Leu88) and C25 of FA throughout the simulations. (**E**) The Gibbs free energy landscape of Wild, MUT-1, MUT-2, and MUT-3. The free energy is expressed in kJ/mol; the colors red and blue represent high and low energy states, respectively.

González-López et al. resolved the Cryo-EM structure of the EF-G and FA complex and analyzed the possible influence of the individual mutation in EF-G through their binding mode. It is important to investigate the difference in the dynamic states of FA in wildtype or mutant EF-G to guide the design of FA derivatives. We performed four independent molecular dynamics (MD) simulations for FA interacting with EF-G, with wildtype or site-mutant proteins. The RMSD, RMSF, Gibbs free energy, MM/PBSA binding free energy, and distance between the α-carbon (Cα) of Phe88 (or Leu88) and FA during the simulation trajectories were compared. As shown in Figure 4A, the RMSD profiles for the wildtype and two mutants had reached equilibrium at approximately 20 ns, except for the MUT-2 system (L461K). The mutant proteins experienced more pronounced conformational changes with high RMSD values during the simulation, consistent with the trends observed in the RMSF, Gibbs free energy, and distance between the key residue and FA (Figure 4B–E). These results corroborate that the interactions of FA with the key residues are essential for FA potency and the stability of the FA-locked conformation.

**Figure 5:**
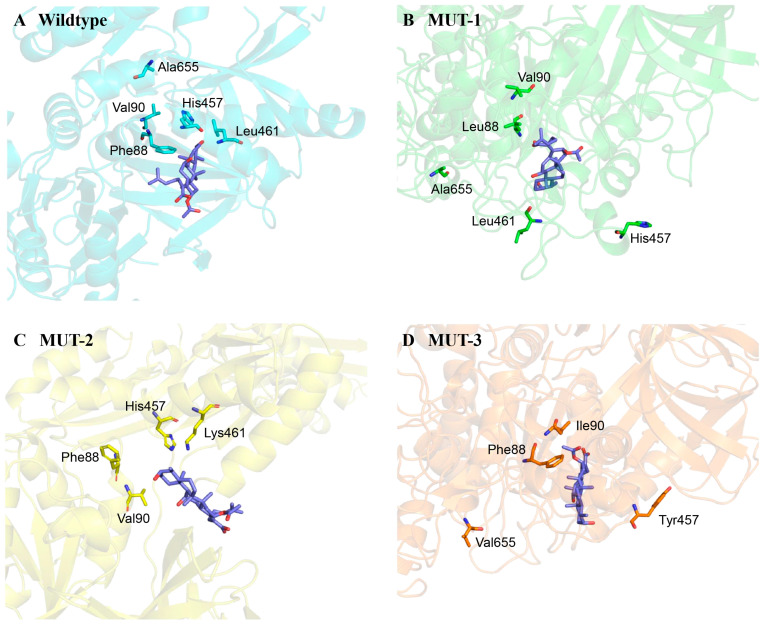
Representative conformations of FA in wildtype and mutant EF-Gs. (**A**) FA (blue stick) with wildtype EF-G (cyan sticks and cartoon); (**B**) FA (blue stick) with MUT-1 (green sticks and cartoon); (**C**) FA (blue stick) with MUT-2 (yellow sticks and cartoon); and (**D**) FA (blue stick) with MUT-3 (orange sticks and cartoon).

MUT-1 vs. wildtype. The wildtype protein (Wild) showed substantially lower RMSD values than MUT-1 during the simulation (Figure 4A). The energy landscape of the wildtype EF-G∙FA complex state showed a clear large global energy minima area (blue in Figure 4E), and the non-blue color in the plot is more prevalent in MUT-1, indicating that MUT-1 seems less stable in comparison with the wildtype. The RMSF of Leu88 in MUT-1 was increased when compared with that of the wildtype (1.37 Å versus 1.28 Å; Figure 4C), and the average distance of Leu88 with FA in the MUT-1 trajectory is longer by 2.5 Å than that of the wildtype complex. This is consistent with the representative conformation observed in Figure 5A,B, which showed that Leu88 in MUT-1 induced conformational flipping of the whole protein and moved FA away from the binding site. These results unequivocally revealed that F88L lost direct contact with FA, resulting in increased drug resistance.

MUT-2 vs. wildtype. L461K belongs to both *fusA* and *fusD* mutations, influencing the stability of EF-G and FA binding [29]. In MUT-2, the L461K mutation induced the system to reach equilibrium after 80 ns, accompanied by high RMSF values of the residues in domain I. Consistently, the distance between F88 and FA showed a more considerable fluctuation than that of the other systems. Compared to the wildtype, MUT-2 showed lower binding free energy with FA (−88.271 KJ/mol for MUT-2 vs. −204.743 KJ/mol for wildtype), indicating the importance of L461 for protein stability and drug binding.

MUT-3 vs. wildtype. Mutations H457Y and V90I in EF-G disrupted the hydrogen-bond network of D434, T436, and H457 and lost hydrophobic contacts with domain III in EF-G; the mutation of A655 with a large-volume valine affected the flexibility of domain V and changed the conformation of the FA binding pocket. In the MUT-3 MD simulation, these three mutations induced a trend similar to the FA binding residue F88L. FA was placed away from F88 in MUT-3 compared to the wildtype (Figure 4D) and showed a significant decrease in binding affinity (−165.827 KJ/mol for MUT-3 vs. −204.743 KJ/mol for wildtype), as confirmed by the few hydrophobic contacts and no hydrogen bonds of FA that interacted with MUT-3 (Figure S1). These results revealed the importance of Val90, His457, and Ala655 for the overall stability of EF-G and the binding with FA.

### 2.3. Property Distributions: Differences Between the Generated Compounds and the Reference Compounds

**Figure 6:**
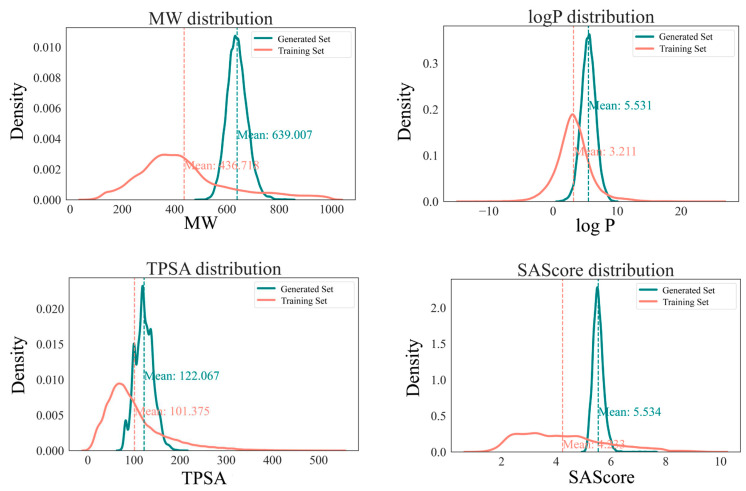
Comparison of property distributions between compounds in the training set (red solid line) and those in the generated set (cyan solid line). The red and cyan dashed lines indicate the mean values of the properties for the compounds in the training set and the generated set, respectively.

The scaffold decorator generated 22,080 FA derivatives based on three types of FA scaffolds. A total of four important properties of the compounds were calculated by RDKit [30], including the lipid and water partition coefficient (LogP), molecular weight (MW), topological polar surface area (TPSA), and synthetic accessibility score (SAScore) [31]. The results indicated that the mean values and overall distribution of the properties of the generated compounds differed significantly from those of the training set due to the diversity of the natural products (Figure 6). Compared to the reference compound FA (MW = 516.35, logP = 5.67, TPSA = 104.06, and SAScore = 5.079), the generated compounds showed a higher MW and TPSA as well as a lower logP, which is considered to be derived from the preference of the polar groups for adding to the FA scaffolds. Notably, the generated compounds displayed a higher SAScore than FA, encouraging us to select the promising derivatives for synthesis.

**Figure 7:**
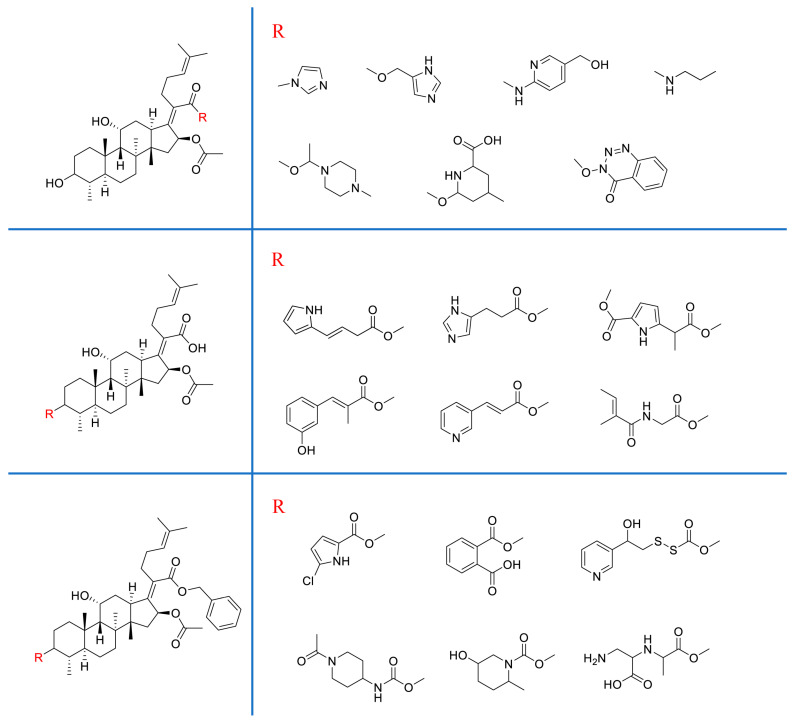
Generated examples.

The examples of the generated sets based on three types of FA scaffolds are shown in Figure 7, indicating that the generated model can selectively grow single bonds, double bonds, and heteroaliphatic or heteroaromatic rings at the attachment sites in the scaffold.

### 2.4. Virtual Screening of the Generated Set

**Figure 8:**
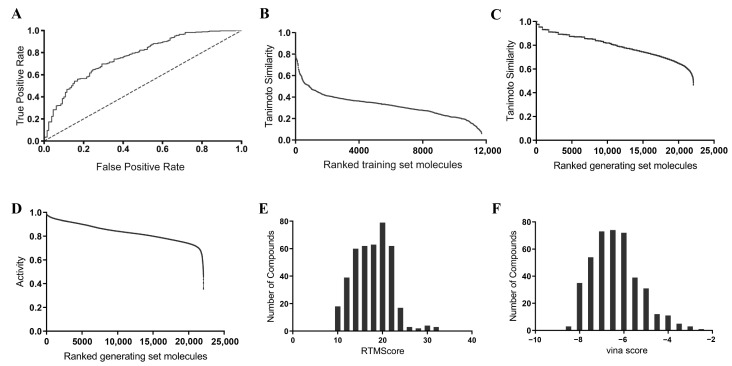
Results of Chemprop and RTMScore. (**A**) ROC-AUC curve of the training set. (**B**,**C**) Tanimoto similarity values between FA and the compounds in the training set (**B**) or the generated set (**C**). (**D**) Score of the generated compounds predicted by Chemprop. (**E**,**F**) Frequency distribution plot of RTMScore (**E**) and vina scores (**F**) for the compounds in the generated set.

To accelerate the hit identification from >20,000 derivatives, we adopted a stepwise method for screening by employing Chemprop and RTMScore [27,28]. For Chemprop, the area under the ROC curve (ROC-AUC) of the test set reached 0.762 ± 0.035, validating the reliability of the model (Figure 8A). The compounds in the training set cover a broad chemical space despite a low similarity with FA (Figure 8B), facilitating the discovery of novel FA derivatives with new features. In contrast, more than 60% of the generated FA derivatives showed a high similarity with FA (similarity value > 0.75) based on the same scaffold (Figure 8C). Then, the anti-MRSA potential of every generated compound was predicted by the Chemprop model, and 409 compounds with a prediction score greater than 0.96 (Figure 8D) were selected for further docking analysis. Among them, 115 compounds showed a higher RTMScore and vina score than FA (RTMScore = 20.3 and vina score = −6.6). Thus, we selected 10 of these compounds with high Chemprop scores and RTMScores, as well as high synthetic accessibility, as determined by visual inspection, for further synthesis and bioactivity evaluation.

### 2.5. Chemistry

**Scheme 1:**
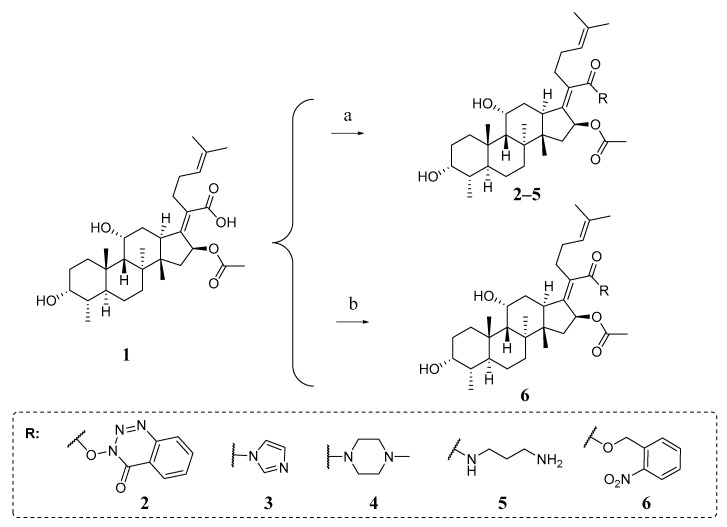
Synthetic route of target compounds **2**–**6.** Reagent and conditions: (**a**) nitrogen-containing heterocyclic or acyclic ammonia, EDCI, CH_2_Cl_2_, r.t.; (**b**) K_2_CO_3_, 2-nitrobenzyl bromide, DMF, 50 °C.

Compounds **2**–**6** were synthesized according to Scheme 1. Firstly, we synthesized derivatives **2**–**5** by using the commercially available FA and structurally modifying its carboxyl group at the C-21 position, which were synthesized by esterification with different alcohols or amines under the catalytic effect of EDCI. Compound **6** was synthesized by substitution reaction between FA and 2-nitrobenzyl bromide under alkaline conditions.

**Scheme 2:**
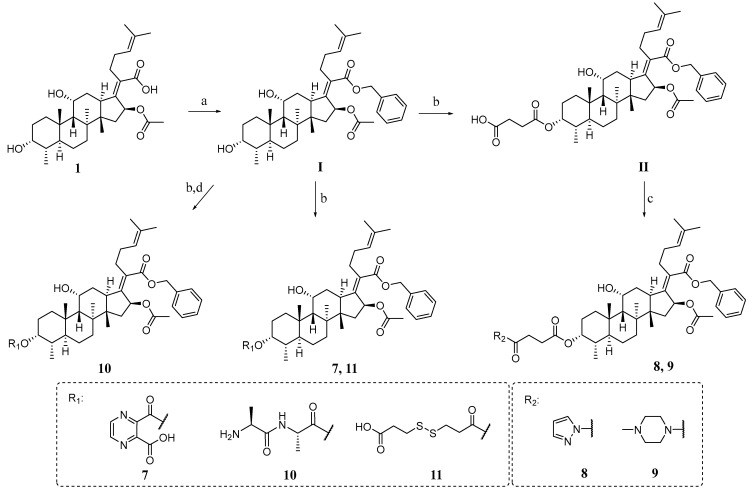
Synthetic route of target compounds **7–11^a^**. **^a^** Reagent and conditions: (a) BrBn, K_2_CO_3_, DMF, 50 °C; (b) anhydrides or acid, DMAP, CH_2_Cl_2_, r.t.; (c) nitrogen-containing heterocyclic ammonia, EDCI, CH_2_Cl_2_, r.t.; and (d) TFA, CH_2_Cl_2_, r.t.

Compounds **7**–**11** were synthesized according to Scheme 2. Intermediate **I** was synthesized by the substitution reaction of its carboxyl group at the C-21 position with BrBn under basic conditions using FA as the starting material. Under DMAP-catalyzed conditions, intermediate **II** was synthesized by esterification of the C-3 position of intermediate **I** with succinic anhydride. Intermediate **II** was amidated with different amines to obtain derivatives **8** and **9**; intermediate **I** was esterified with different acids to obtain compounds **7** and **11**. Intermediate **I** was reacted with N-Boc-L-alanyl-L-alanine by amidation and further removal of the Boc-protecting group to obtain derivative **10**. The chemical structures of all the derivatives were structurally confirmed by ^1^H-NMR, ^13^C-NMR, and HR-MS.

### 2.6. Structure–Activity Relationship of FA Derivatives

**Figure 9:**
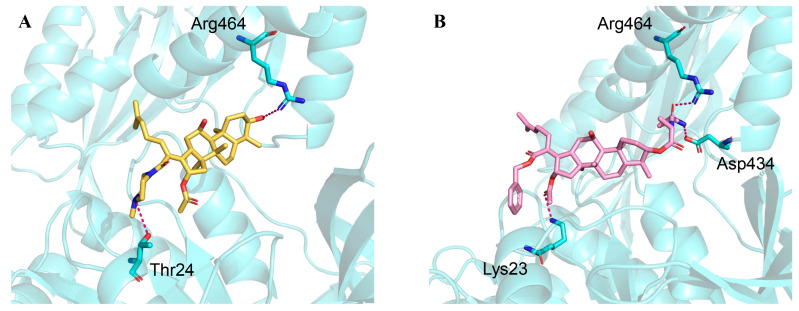
The binding modes of compounds **4** ((**A**)**,** golden) or **10** ((**B**), pink) with EF-G (cyan). The hydrogen bonds are shown with red dotted lines.

Five derivatives modified at the 21-COOH position of FA were synthesized, and the anti-MRSA activities of five derivatives were assessed with the inhibition rates and minimum inhibitory concentrations (MIC_90_; Table 1). Compound **4** demonstrated the most potent anti-MRSA activity (MIC = 16 μg/mL) because of the piperazine ring forming a hydrogen bond with Thr24 (Figure 9). Compound **5** exhibited a slight decrease in anti-MRSA activity, potentially due to a conformational flexibility in its acyclic amine moiety to restrict the binding to Thr24. In contrast, compound **3** was less active, because the N...N distance in the imidazole ring is significantly shorter than that in the piperazine group (**4**) to destroy the hydrogen bond with Thr24. Replacing the hydroxyl group in FA with 3-hydroxybenzo[d][1,2,3]triazin-4(3H)-one (**2**) or 2-Nitrobenzyl alcohol (**6**) had an adverse effect on the MIC_90_ value. These results clearly indicate that the volume and properties of the amine substitutions at the 21-position are critical for maintaining anti-MRSA activity.

For the modification at the 3-position (Table 2), the replacement with L-alanyl-L-alanine (10) yielded the most potent compound against MRSA (MIC_90_ = 16 μg/mL), which may be attributed to the formation of two hydrogen bonds between the L-alanyl-L-alanine chain of 10 and Arg464 and Asp434 (Figure 9). In contrast, replacement with heteroaliphatic or heteroaromatic nitrogens (**7**–**9**) demonstrated 2–8-fold decreases in potency compared to compound **10**. This manifested that the substitution at the 3-position containing an alkyl chain bearing basic nitrogen atoms is optimal to the compounds for maintaining anti-MRSA activity.

**Figure 10:**
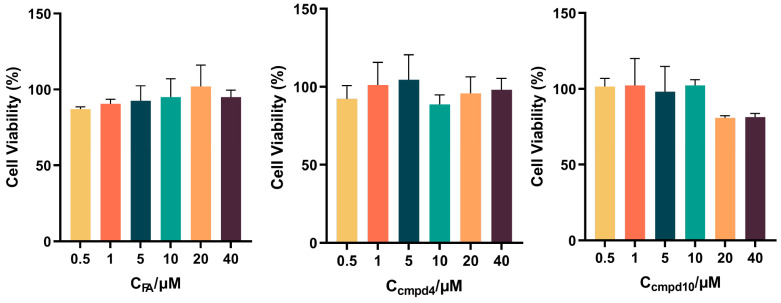
Cytotoxic effect of compounds (FA, compounds **4** and **10**) on cell viability in 293T cells. The cell viability was evaluated by MTT assay.

We tested the cell viability of compounds **4** and **10** in the concentration range of 0.5 to 40 μM using the MTT assay (Figure 10). The results showed that compound **4** maintained nearly 100% cell viability at 40 μM, similar to FA. The cell viability of compound **10** was also above 80%. The cytotoxicity assay results showed that the cell viability of compounds **4** and **10** was >70% of the negative control, indicating a nontoxic range as defined in ISO 10993-5:2009(E) [32].

## 3. Materials and Methods

### 3.1. Homology Modeling of Wildtype and Mutant EF-Gs from S. Aureus

At the outset of this work, the complex of EF-G bound to FA had not been published, so we downloaded the prediction structure of wildtype EF-G from the AlphaFold DB [33,34].

To investigate the mechanism of FA resistance mutations in EF-G, we performed amino acid mutations on the 3D structure of EF-G. The *fusA* and *fusD* mutants in EF-G are critical subtypes that influence the stability of EF-G and FA binding and decrease FA affinity with EF-G [29]. In light of this, we constructed three mutants at EF-G:

1. Mutant 1 (MUT-1) with F88L in EF-G. The mutation of Phe88 in EF-G significantly affected its structure and function, which was considered a high-level resistant mutation (FA: MIC ≥ 64 μg/mL) [18,35].
2. Mutant 2 (MUT-2) with L461K in EF-G. FA showed no directed interactions with EF-G in the Cryo-EM complex [36]. However, the L461K alteration was one of the most common FA resistance determinants, belonging to *fusA* and *fusD* mutations, which influence FA binding and EF-G stability [37,38,39]. L461 has been confirmed to lead to high-level resistance of FA with MIC > 256 μg/mL in clinical strains of *S. aureus* [18,37].
3. Mutant 3 (MUT-3). This type involves three mutations, V90I, H457Y, and A655V, which are common mutant sites inducing FA resistance [29,38], especially H457Y with an abolished capability of FA inhibiting EF-G (MIC ≥ 512 μg/mL) [39].

### 3.2. Molecular Docking

The binding modes of FA and different types of EF-Gs were identified using the Surflex-Dock module in SYBYL-X (version 2.1.1, Tripos Inc.). The 3D structures of FA and the receptors were first hydrogenated using SYBYL-X to ensure proper protonation states, followed by charge optimization with the Gasteiger–Hückel (ligand) and Gasteiger–Marsili (receptor) methods. The active site was set around the key residues Phe88, Asp434, Thr436, Leu456, His457, and Arg464 [22,29,36]. Other parameters were kept to the default throughout the simulation.

### 3.3. Molecular Dynamics (MD) Simulation

Four independent simulations were performed for FA interacting with different types of EF-Gs to further investigate the influence of the mutant residues on the binding of the EF-G∙FA complex. The initial coordinates of EF-G and FA were derived from the previously mentioned docking conformations. Taking the wildtype EF-G∙FA simulation as an example, the GAFF force field [40] was applied for FA using the Sobtop 1.0 software [41], and the receptor was modeled with the Amber99SB force field [42] in GROMACS 2022.5 [43]. The system was solvated using the TIP3P waters and neutralized with 0.15 mol/L NaCl. Then, energy minimization was performed for 100,000 steps using the steep-descent minimization integrator. The canonical ensemble was carried out by heating the system from 0 K to 300 K using a velocity-scaling thermostat [44] and equilibrated for 2 ns at 300 K. The isothermal–isobaric ensemble (P = 1 bar and T = 300 K) was constructed using the Parrinello–Rahman barostat [45,46] and equilibrated for 10 ns. Finally, a full-atom simulation of 100 ns was performed for the system. All the bonds with hydrogen atoms were constrained with the linear constraint solver (LINCS) algorithm [47], and the long-range electrostatics were treated with the Particle Mesh Ewald method [48].

The root mean square deviation (RMSD), root mean square fluctuation (RMSF), distance, and Gibbs free energy were calculated to evaluate the stability of the EF-G·FA complex. The GROMOS clustering algorithm [49] was used to cluster the simulation trajectories of the system based on the RMSD and extracted the representative conformation of EF-G·FA for an interaction analysis by LigPlot [50]. MM/PBSA binding free energy calculations were performed with a 90–100 ns interval in the MD trajectories using a modified gmx_mmpbsa script (available at https://github.com/Jerkwin/gmxtools/tree/master/gmx_mmpbsa, accessed on 28 March 2024).

### 3.4. Scaffold Generator-Decorator

**Figure 11:**
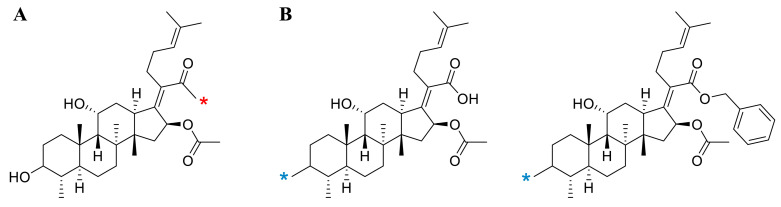
Scaffolds for the molecular generator were modified at the 21- (**A**; red) or 3-positions (**B**; blue) of FA. “*” means the generation site on the scaffold.

In this study, novel FA derivatives were generated by a scaffold decorator based on the framework of Recurrent Neural Networks [51]. First, 407,270 compounds were downloaded from the COlleCtion of Open NatUral producTs (COCONUT) database [49], before eliminating unreadable compounds. The REtrosynthetic Combinatorial Analysis Procedure (RECAP) [52] was utilized, extracting 311,066 scaffold-decoration tuples from the compounds. The randomized SMILES representations [53] with 5-folds were performed for the scaffold-decoration dataset. Then, the model was trained with a batch size of 256, a learning rate start of 0.001, and a learning rate min of 0.0001, and the scaffolds modified at the 3- or 21-positions of FA (Figure 11) were fed in manually. Finally, the model generated 22,080 novel FA derivatives by adding the decoration groups to the attachment points on the FA scaffolds.

### 3.5. Chemprop

A total of 2328 compounds against the keyword ‘Staphylococcus’ with minimum inhibitory concentration (MIC) values were retrieved from the PubChem database (https://pubchem.ncbi.nlm.nih.gov/, accessed on 15 June 2024). To expand the chemical space of the model, we employed the randomized SMILES method [53] to represent the compounds in the dataset, resulting in a total of 11,472 samples. These samples were classified into two categories, including 8124 positive samples (MIC ≤ 4 μM) and 3348 negative samples (MIC > 4 μM), according to the previous study [54]. Then, this dataset was divided into the training, validation, and test sets using the ratio of 8:1:1.

The core of the Chemprop model is the directed message-passing neural network (D-MPNN) [55], in which messages are transmitted through directed chemical bonds (edges) rather than atoms, thereby improving the performance and accuracy of the model [27]. With the settings (MPNN depth = 6, dropout rate = 0.2, and number of neurons in the feed-forward layers = 1600), a binary classification model was conducted to predict the potential anti-MRSA activity of the generated compounds. Among them, 409 compounds with predicted scores > 0.96 were selected for subsequent screening.

### 3.6. RTMScore

RTMScore is a scoring function developed to analyze protein–ligand interactions while concurrently assessing their binding affinities by deriving residue–atom distance likelihoods through a mixture density network [28]. Here, we utilized the representative conformations of wildtype EF-G and FA obtained from the MD simulation to predict the binding affinities of the compounds screened from Chemprop. The binding conformation and scores of the compounds were recorded.

### 3.7. Detailed Synthetic Procedure

The chemicals and reagents were analytically pure or dried with standard methods when necessary. The progress of all the reactions was monitored by thin-layer chromatography (TLC) on silica gel HSGF254 (Qingdao, China) using fluorescence with a wavelength of 254 nm on a ZF7-C three-purpose UV analyzer (Shanghai, China) and 10% ethanol sulfate solution (Shanghai, China) to detect the spots. All the synthesized compounds were purified by column chromatography on silica gel. ^1^H NMR (400 MHz) and ^13^C NMR (100 MHz) spectra were measured by a Bruker av400 (Bruker, German) instrument using CDCl_3_ as the solvent and TMS as the internal standard. Hertz expresses chemical shifts in δ values (ppm) and the coupling constants (*J*). High-resolution mass spectra (HRMS) were recorded on an Agilent QTOF 6520 or 6530 spectrometer (Waldbronn, German). Unless otherwise specified, all the reagents used in the synthesis are sourced from Shanghai, China. 

#### 3.7.1. General Procedure for the Synthesis of 1–6

Fusidic acid (**1**)

Fusidic acid was purchased from Guangzhou Yibang Pharmaceutical Company and was obtained in a 100 g size with a purity of 99%. This thesis confirmed its purity and structure in the preliminary study. A 1 mg sample of fusidic acid, with the appearance of white powder, was weighed and dissolved in ethyl acetate. Its purity was examined using TLC, using three different unfolding systems (*V*_chloroform_:*V*_methanol_ = 10:1, *V*_cyclohexane_:*V*_ethyl acetate_ = 1:1, and *V*_petroleum ether_:*V*_acetone_ = 3:1) for the fusidic acid sample to unfold the fusidic acid samples. A spot was shown by UV color development with ethanol sulfate. From this, it was generally determined that the purchased sample of fusidic acid was of high purity. The melting point of the fusidic acid sample was determined to be 190.1–192.3 °C using a melting point apparatus, which is consistent with the theoretical reference value.

4-oxobenzo[d][1,2,3]triazin-3(4H)-yl(Z)-2-((3R,4S,5S,8S,9S,10S,11R,13R,14S,16S)-16-acetoxy-3,11-dihydroxy-4,8,10,14-tetramethylhexadecahydro-17H-cyclopenta[a]phenanthren-17-ylidene)-6-methylhept-5-enoate (**2**).

Fusidic acid (206.7 mg, 0.4 mmol) was dissolved in anhydrous dichloromethane (20.0 mL), and EDCI (134.2 mg, 0.7 mmol) and 3-hydroxybenzo[d][1,2,3]triazin-4(3H)-one (146.8 mg, 0.9 mmol) were added sequentially. The reaction was stirred at room temperature for 5 h. At the end of the reaction, it was diluted by adding dichloromethane. The mixture was washed sequentially with water and saturated sodium chloride solution, dried with anhydrous Na_2_SO_4_, and concentrated under reduced pressure. The residue was purified by silica gel column chromatography, using trichloromethane-methanol (150:1~50:1) as the eluent, to obtain compound **2** (220 mg; yield 78.1%). m.p. 214.7–217.6 °C. R_f_ = 0.3 (dichloromethane/methanol = 20:1). ^1^H NMR (400 MHz, CDCl_3_): δ = 8.34 (dd, *J* = 9.0 Hz, 1H, Ar-H), 8.19 (d, *J* = 7.9 Hz, 1H, Ar-H), 8.03–7.93 (m, 1H, Ar-H), 7.81 (m, 1H, Ar-H), 5.92 (d, *J* = 8.4 Hz, 1H, 16-H), 5.18 (t, *J* = 7.2 Hz, 1H, 24-H), 4.37 (s, 1H, 11-H), 3.75 (s, 1H, 3-H), 3.14 (d, *J* = 12.4 Hz, 1H, 13-H), 2.75–2.56 (m, 2H, 2×22-H), 2.43–2.30 (m, 3H, 1-H, 5-H,12-H), 2.29–2.10 (m, 3H, 15-H and 2×23-H), 2.07 (s, 3H, -OCOCH_3_), 1.80–1.70 (m, 2H, 2-H and 12-H), 1.68 (s, 3H, 27-CH_3_), 1.64 (s, 3H, 26-CH_3_), 1.38 (s, 3H, 30-CH_3_), 1.23 (s, 3H, 19-CH_3_), 1.17–1.05 (m, 2H, 6-H and 7-H), 0.97 (s, 3H, 18-CH_3_), 0.92 (s, 3H, 28-CH_3_). ^13^C NMR (100 MHz, CDCl_3_): δ = 170.99, 165.28, 161.77, 150.28, 144.34, 135.21, 133.00, 132.52, 128.83, 125.68, 125.61, 122.72, 122.39, 74.25, 71.31, 68.13, 49.13, 48.87, 45.07, 39.44, 39.00, 37.04, 36.14, 36.13, 35.50, 32.38, 30.24, 29.90, 28.97, 28.37, 25.74, 24.22, 22.71, 21.09, 20.68, 18.05, 17.84, 15.89. HRMS-ESI *m*/*z*: calcd for C_38_H_51_N_3_O_7_ [*M*+Na]^+^: 684.3619, found: 684.3607.

(3R,4S,5S,8S,9S,10S,11R,13R,14S,16S,Z)-17-(1-(1H-imidazol-1-yl)-6-methyl-1-oxohept-5-en-2-ylidene)-3,11-dihydroxy-4,8,10,14-tetramethylhexadecahydro-1H-cyclopenta[a]phenanthren-16-ylacetate (**3**).

Referring to the synthesis of compound **2**, compound **1** (206.7 mg, 0.4 mmol) was reacted with imidazole (61.23 mg, 0.9 mmol), and the residue was purified by silica gel column chromatography using trichloromethane-methanol (120:1~30:1) as the eluent, to obtain compound **3** (174 mg; yield 76.9%). m.p. 208.1–210.5 °C. R_f_ = 0.2 (dichloromethane/methanol = 20:1). ^1^H NMR (400 MHz, CDCl_3_): δ = 8.07 (s, 1H, IMZ-H), 7.43 (s, 1H, IMZ-H), 7.10 (s, 1H, IMZ-H), 5.48 (d, *J* = 8.7 Hz, 1H, 16-H), 5.01 (t, *J* = 7.2 Hz, 1H, 24-H), 4.39 (s, 1H, 11-H), 3.76 (s, 1H, 3-H), 3.13 (d, *J* = 10.8 Hz, 1H, 13-H), 2.20–2.10 (m, 3H, 2×22-H and 1-H), 1.90–1.75 (m, 7H, 2-H, 5-H, 2×12-H, 15-H and 2×23-H), 1.63 (s, 3H, -OCOCH_3_), 1.54 (s, 6H, 27-CH_3_ and 26-CH_3_), 1.39 (s, 3H, 30-CH_3_), 1.25 (s, 3H, 19-CH_3_), 1.15–1.05 (m, 2H, 6-H and 7-H), 0.98 (s, 3H, 18-CH_3_), 0.93 (s, 3H, 28-CH_3_). ^13^C NMR (100 MHz, CDCl_3_,): δ = 171.15, 169.28, 149.28, 138.68, 134.99, 131.99, 131.31, 123.48, 117.60, 75.32, 72.70, 69.41, 50.82, 50.56, 44.79, 40.89, 40.21, 38.41, 37.72, 37.39, 36.50, 33.62, 31.62, 31.28, 31.09, 30.75, 29.08, 27.07, 25.38, 24.39, 22.18, 21.17, 19.27, 17.32. HRMS-ESI *m*/*z*: calcd for C_34_H_50_N_2_O_5_ [*M*+Na]^+^: 589.3612, found: 589.3599.

(3R,4S,5S,8S,9S,10S,11R,13R,14S,16S,Z)-3,11-dihydroxy-4,8,10,14-tetramethyl-17-(6-methyl-1-(4-methylpiperazin-1-yl)-1-oxohept-5-en-2-ylidene)hexadecahydro-1H-cyclopenta[a]phenanthren-16-ylacetate (**4**).

Referring to the synthesis of compound **2**, compound **1** (206.7 mg, 0.4 mmol) was reacted with N-methylpiperazine (90.0 mg, 0.9 mmol), and the residue was purified by silica gel column chromatography using trichloromethane-methanol (120:1~50:1) as the eluent, to obtain compound **4** (173 mg; yield 72.5%). m.p. 203.5–208.5 °C. R_f_ = 0.1 (dichloromethane/methanol = 20:1). ^1^H NMR (400 MHz, CDCl_3_): δ = 5.49 (d, *J* = 8.6 Hz, 1H, 16-H), 5.08 (t, *J* = 7.2 Hz, 1H, 24-H), 4.36 (s, 1H, 11-H), 3.76 (s, 1H, 3-H), 3.70–3.20 (m, 4H, PIP-H), 3.05–2.87 (m, 3H, PIP-H and 13-H), 2.80–2.36 (m, 6H, PIP-H), 2.35 (s, 1H, PIP-CH_3_), 2.32–2.25 (m, 2H, 2×22-H), 2.23–2.05 (m, 6H, 1-H, 5-H, 12-H, 15-H and 2×23-H), 2.00 (s, 3H, -OCOCH_3_), 1.93–1.83 (m, 2H, 2-H and 12-H), 1.69 (s, 3H, 27-CH_3_), 1.61 (s, 6H, 26-CH_3_ and 30-CH_3_), 1.40 (s, 3H, 19-CH_3_), 1.17–1.05 (m, 2H, 6-H and 7-H), 0.98 (s, 3H, 18-CH_3_), 0.92 (s, 3H, 28-CH_3_). ^13^C NMR (100 MHz, CDCl_3_): δ = 172.21, 171.75, 143.39, 141.48, 133.77, 124.76, 76.08, 74.46, 72.73, 69.83, 69.60, 56.50, 55.57, 50.68, 50.46, 47.21, 44.36, 41.99, 40.85, 38.50, 37.60, 36.92, 33.87, 31.78, 31.61, 31.37, 29.18, 27.10, 25.61, 25.36, 24.13, 22.61, 22.16, 19.34, 19.17, 17.35. HRMS-ESI *m*/*z*: calcd for C_36_H_58_N_2_O_5_ [*M*+Na]^+^: 621.4238, found: 621.4308.

(3R,4S,5S,8S,9S,10S,11R,13R,14S,16S,Z)-17-(1-((3-aminopropyl)amino)-6-methyl-1-oxohept-5-en-2-ylidene)-3,11-dihydroxy-4,8,10,14-tetramethylhexadecahydro-1H-cyclopenta[a]phenanthren-16-ylacetate (**5**).

Referring to the synthesis of compound **2**, compound **1** (206.7 mg, 0.4 mmol) was reacted with propane-1,3-diamine (66.7 mg, 0.9 mmol), and the residue was purified by silica gel column chromatography using trichloromethane-methanol (100:1~30:1) as the eluent, to obtain compound **5** (137 mg; yield 60.1%). m.p. 76.2–79.7 °C. R_f_ = 0.1 (dichloromethane/methanol = 20:1). ^1^H NMR (400 MHz, CDCl_3_): δ = 9.13 (d, *J* = 6.6 Hz, 1H, -CONH-), 5.61 (d, *J* = 8.8 Hz, 1H, 16-H), 5.08 (ddt, *J* = 8.7, 5.6, 1.5 Hz, 1H, 24-H), 4.39–4.33 (m, 1H, 11-H), 3.92–3.57 (m, 3H, 3-H, -NCH_2_-), 3.40–3.22 (m, 2H, -NCH_2_-), 3.03 (d, *J* = 11.2 Hz, 1H, 13-H), 1.91 (s, 3H, -OCOCH_3_), 1.67 (d, *J* = 1.4 Hz, 3H, -CH_3_), 1.60 (d, *J* = 1.4 Hz, 3H, -CH_3_), 1.38 (s, 3H, -CH_3_), 0.98 (s, 3H, -CH_3_), 0.94–0.89 (m, 6H, 2×-CH_3_). ^13^C NMR (100 MHz, CDCl_3_): δ = 170.07, 155.01, 143.16, 133.15, 132.55, 122.57, 71.39, 68.06, 56.55, 49.71, 49.50, 43.97, 42.69, 39.55, 39.10, 37.10, 36.42, 36.11, 35.25, 34.94, 32.35, 30.26, 30.04, 29.76, 29.38, 27.98, 25.83, 24.00, 23.02, 20.90, 18.11, 17.97, 16.03, 14.91. HRMS-ESI *m*/*z*: calcd for C_34_H_56_N_2_O_5_ [*M*+Na]^+^: 595.4081, found: 595.3810.

2-nitrobenzyl(Z)-2-((3R,4S,5S,8S,9S,10S,11R,13R,14S,16S)-16-acetoxy-3,11-dihydroxy-4,8,10,14-tetramethylhexadecahydro-17H-cyclopenta[a]phenanthren-17-ylidene)-6-methylhept-5-enoate (**6**).

Fusidic acid (220 mg, 0.4 mmol) was dissolved in DMF (20 mL), and potassium carbonate (110.6 mg, 0.8 mmol) and 2-nitrobenzyl bromide (150.5 mg, 0.7 mmol) were added. The reaction was stirred at 50 °C for 5 h. At the end of the reaction, the reaction mixture was diluted by adding ethyl acetate; the mixture was washed sequentially with water and saturated sodium chloride solution, dried with anhydrous Na_2_SO_4_, and concentrated under reduced pressure; and the residue was purified by silica gel column chromatography, using petroleum ether-ethyl acetate (100:1~10:1) as the eluent, to obtain compound **6** (214.6 mg; yield 82.3%). m.p. 79.6–84.2 °C. R_f_ = 0.4 (dichloromethane/methanol = 20:1). ^1^H NMR (400 MHz, CDCl_3_): δ = 8.08 (dd, *J* = 8.2, 1.2 Hz, 1H, Ar-H), 7.65–7.56 (m, 2H, Ar-H), 7.46 (td, *J* = 7.8, 7.0, 1.9 Hz, 1H, Ar-H), 5.89–5.84 (m, 1H, 16-H), 5.65 (d, *J* = 14.8 Hz, 1H, -CHAr), 5.23 (d, *J* = 14.8 Hz, 1H, -CHAr), 5.06 (t, *J* = 7.8 Hz, 1H, 24-H), 4.32 (s, 1H, 11-H), 3.71 (d, *J* = 2.5 Hz, 1H, 3-H), 3.05 (d, *J* = 10.5 Hz, 1H, 13-H), 2.53–2.37 (m, 2H, 2×22-H), 2.30 (d, *J* = 13.1 Hz, 1H, 12-H), 2.18–1.98 (m, 5H,15-H, 2×23-H, 1-H and 5-H), 1.92 (s, 3H, -OCOCH_3_), 1.61 (s, 3H, 27-CH_3_), 1.52 (s, 3H, 26-CH_3_), 1.35 (s, 3H, 30-CH_3_), 0.95 (s, 3H, 19-CH_3_), 0.89 (d, *J* = 6.9 Hz, 6H, 18-CH_3_, 28-CH_3_). ^13^C NMR (100 MHz, CDCl_3_): δ = 170.40, 169.24, 149.86, 147.53, 133.75, 132.58, 132.14, 129.71, 129.24, 128.75, 125.07, 123.01, 74.37, 71.39, 68.21, 62.85, 49.31, 48.71, 44.23, 39.47, 39.04, 36.92, 36.41, 35.91, 35.53, 32.11, 30.16, 29.91, 28.98, 28.53, 25.71, 23.94, 23.03, 20.95, 20.87, 17.82, 17.76, 15.96. HRMS-ESI *m*/*z*: calcd for C_38_H_53_NO_8_ [*M*+Na]^+^: 674.3663, found: 674.3644.

#### 3.7.2. General Procedure for the Synthesis of 7–11

3-((((3R,4S,5S,8S,9S,10S,11R,13R,14S,16S,Z)-16-acetoxy-17-(1-(benzyloxy)-6-methyl-1-oxohept-5-en-2-ylidene)-11-hydroxy-4,8,10,14-tetramethylhexadecahydro-1H-cyclopenta[a]phenanthren-3-yl)oxy)carbonyl)pyrazine-2-carboxylic acid (**7**).

Fusidic acid (5.0 g, 9.7 mmol) was dissolved in DMF (100 mL), K_2_CO_3_ (2.7 g, 19.5 mmol) and benzyl bromide (1.4 mL, 11.5 mmol) were added, and the reaction was stirred for 5 h at 50 °C. At the end of the reaction, the reaction mixture was diluted by adding ethyl acetate; the mixture was washed sequentially with water and saturated sodium chloride solution, dried with anhydrous Na_2_SO_4_, and concentrated under reduced pressure; and the residue was purified by silica gel column chromatography, using trichloromethane-methanol (200:1~80:1) as the eluent, to obtain Intermediate **I** (4.4 g, 75.4%). Intermediate **I** (424.8 mg, 0.7 mmol) was dissolved in anhydrous dichloromethane (20 mL), 2,3-pyrazine dicarboxylic anhydride (525.3 mg, 3.5 mmol) and DMAP (256.6 mg, 2.1 mmol) were added, and the reaction was stirred at room temperature for 10 h. At the end of the reaction, the mixture was diluted with dichloromethane and washed twice with 5% HCl solution, deionized water, and a saturated sodium chloride solution. It was then dried over anhydrous Na_2_SO_4_ and concentrated under reduced pressure. The residue was purified by silica gel column chromatography, using trichloromethane-methanol (150:1~30:1) as the eluent to obtain compound **7** (387.9 mg; yield 73.2%). m.p. 232.0–234.1 °C. R_f_ = 0.2 (dichloromethane/methanol = 20:1). ^1^H NMR (400 MHz, CDCl_3_): δ = 8.69–8.72 (m, 2H, 2×Ar-H), 7.32–7.36 (m, 5H, 5×Ar-H), 5.84 (d, *J* = 7.76 Hz, 1H, 16-H), 5.71 (s, 1H, 11-OH), 5.23 (s, 1H, 11-H), 5.17 (d, *J* = 12.07 Hz, 1H, -CHAr), 5.02 (t, *J* = 7.11 Hz, 1H, 24-H), 4.94 (d, *J* = 12.12 Hz, 1H, -CHAr), 3.18 (s, 1H, 3-H), 2.95 (d, *J* = 10.98 Hz, 1H, 13-H), 2.63–2.66 (m, 3H, 12-H and 2×22-H), 2.36–2.46 (m, 5H, 1-H, 5-H, 15-H and 2×23-H), 2.05–2.11 (m, 4H, 2×2-H, 7-H and 12-H), 1.92 (s, 3H, -OCOCH_3_), 1.79–1.83 (m, 4H, 1-H, 4-H, 6-H and 9-H), 1.54 (s, 3H, 27-CH_3_), 1.43 (s, 3H, 26-CH_3_), 1.26–1.31 (m, 1H, 15-H), 1.19 (s, 3H, 30-CH_3_), 1.11–1.14 (m, 2H, 6-H and 7-H), 1.05 (s, 3H, 19-CH_3_), 0.96 (s, 3H, 18-CH_3_), 0.87 (d, *J* = 4.40 Hz, 3H, 28-CH_3_). ^13^C NMR (100 MHz, CDCl_3_): δ = 170.55, 169.98, 166.73, 165.01, 164.53, 147.50, 146.13, 145.92, 144.82, 144.57, 135.57, 132.71, 131.01, 128.59, 128.35, 122.92, 77.75, 74.22, 73.94, 66.52, 39.35, 38.84, 37.84, 37.74, 36.72, 34.63, 32.81, 32.24, 32.13, 30.09, 30.02, 29.69, 28.99, 28.20, 26.35, 25.61, 24.16, 24.11, 22.36, 20.92, 20.29, 18.19, 17.58, 15.38. HRMS-ESI *m*/*z*: calcd for C_44_H_56_N_2_O_9_ [*M*+Na]^+^: 779.3873 found: 779.3854.

benzyl(Z)-2-((3R,4S,5S,8S,9S,10S,11R,13R,14S,16S)-16-acetoxy-11-hydroxy-4,8,10,14-tetramethyl-3-((4-oxo-4-(1H-pyrazol-1-yl)butanoyl)oxy)hexadecahydro-17H-cyclopenta[a]phenanthren-17-ylidene)-6-methylhept-5-enoate (**8**).

Intermediate **I** (424.8 mg, 0.7 mmol) was dissolved in anhydrous dichloromethane (20 mL), succinic anhydride (350.2 mg, 3.5 mmol) and DMAP (256.6 mg, 2.1 mmol) were added, and the reaction was stirred at room temperature for 10 h. At the end of the reaction, the solution was diluted with dichloromethane; washed twice each with 5% HCl solution, deionized water, and a saturated sodium chloride solution; and then dried over anhydrous Na_2_SO_4_ and concentrated under reduced pressure. The residue was purified by silica gel column chromatography, using trichloromethane-methanol (130:1~30:1) as the eluent to obtain intermediate **II** (411.7 mg, 83.2%). Intermediate **II** (353.5 mg, 0.5 mmol) was dissolved in anhydrous dichloromethane (50 mL), pyrazole (68.1 mg, 1.0 mmol) and EDCI (149.5 mg, 0.78 mmol) were added, and the reaction was stirred for 6 h at room temperature. At the end of the reaction, dichloromethane was added to dilute it; the mixture was washed with water, washed with saturated sodium chloride solution, dried with anhydrous Na_2_SO_4_, and concentrated under reduced pressure; and the residue was purified by silica gel column chromatography, using trichloromethane-methanol (150:1~50:1) as the eluent, to obtain compound **8** (282.7 mg; yield 74.7%). m.p. 233.4–236.0 °C. R_f_ = 0.5 (dichloromethane/methanol = 20:1). ^1^H NMR (400 MHz, CDCl_3_): δ = 8.26 (d, *J* = 2.60 Hz, 1H, pyr-H), 7.73 (s, 1H, pyr-H), 7.41–7.30 (m, 5H, 5×Ar-H), 6.46 (dd, *J* = 2.8, 1.5 Hz, 1H, pyr-H), 5.90 (d, *J* = 8.30 Hz, 1H, 16-H), 5.23 (d, *J* = 12.20 Hz, 1H, -CHAr), 5.08 (t, *J* = 7.20 Hz, 1H, 24-H), 4.99 (d, *J* = 2.40 Hz, 1H, 11-OH), 4.94 (d, *J* = 12.20 Hz, 1H, -CHAr), 4.35 (s, 1H, 11-H), 3.54–3.47 (m, 2H, -CH_2_-), 3.02 (d, *J* = 11.10 Hz, 1H, 13-H), 2.88–2.81 (m, 2H,-CH_2_-), 2.56–2.40 (m, 2H, 2×22-H), 2.35–2.27 (m, 1H, 12-H), 2.23–2.00 (m, 5H, 15-H, 2×23-H, 1-H and 5-H), 1.95 (s, 3H, -OCOCH_3_), 1.90–1.79 (m, 3H, 2×2-H and 12-H), 1.66 (s, 3H, 27-CH_3_), 1.63–1.56 (m, 1H, 7-H), 1.55 (s, 3H, 26-CH_3_), 1.28 (s, 3H, 30-CH_3_), 1.18–1.02 (m, 2H, 6-H and 7-H), 0.98 (s, 3H, 19-CH_3_), 0.92 (s, 3H, 18-CH_3_), 0.84 (s, 3H, 28-CH_3_). ^13^C NMR (100 MHz, CDCl_3_): δ = 171.69, 171.06, 170.56, 170.00, 148.49, 144.24, 135.83, 132.61, 130.50, 128.64, 128.58, 128.44, 128.34, 123.12, 109.75, 74.90, 74.47, 68.24, 66.48, 49.16, 48.76, 44.02, 39.45, 39.08, 37.81, 36.92, 34.96, 32.60, 31.01, 30.65, 29.56, 29.09, 28.91, 28.41, 27.31, 25.79, 24.27, 22.75, 21.03, 20.67, 19.26, 18.00, 17.80, 15.64, 13.81. HRMS-ESI *m*/*z*: calcd for C_45_H_60_N_2_O_8_ [*M*+Na]^+^: 779.4242, found: 779.4227.

benzyl(Z)-2-((3R,4S,5S,8S,9S,10S,11R,13R,14S,16S)-16-acetoxy-11-hydroxy-4,8,10,14-tetramethyl-3-((4-(4-methylpiperazin-1-yl)-4-oxobutanoyl)oxy)hexadecahydro-17H-cyclopenta[a]phenanthren-17-ylidene)-6-methylhept-5-enoate (**9**).

Referring to the synthesis of compound **8**, Intermediate **II** (353.5 mg, 0.5 mmol) was reacted with N-methylpiperazine (100.2 mg, 1.0 mmol), and the residue was purified by silica gel column chromatography using trichloromethane-methanol (120:1~50:1) as the eluent, to obtain compound **9** (286.8 mg; yield 72.7%). m.p. 224.7–226.9 °C. R_f_ = 0.2 (dichloromethane/methanol = 20:1). ^1^H NMR (400 MHz, CDCl_3_): δ = 7.43–7.31 (m, 5H, 5×Ar-H), 5.90 (d, *J* = 8.40 Hz, 1H, 16-H), 5.23 (d, *J* = 12.20 Hz, 1H, -CHAr), 5.08 (t, *J* = 7.10 Hz, 1H, 24-H), 4.98–4.91 (m, 2H, -CHAr and 11-OH), 4.33 (s, 1H, 11-H), 3.73–3.48 (m, 4H, -CON(CH_2_)_2_), 3.04 (d, *J* = 11.60 Hz, 1H, 13-H), 2.75–2.61 (m, 4H, 2×-CH_2_-), 2.51–2.35 (m, 7H, -N(CH_2_)_2_, 2×22-H and 12-H), 2.32 (s, 3H, -NCH_3_), 2.25–1.98 (m, 6H, 15-H, 2×23-H, 1-H, 5-H and 12-H), 1.95 (s, 3H, -OCOCH_3_), 1.90–1.67 (m, 6H, 2×2-H, 1-H, 4-H, 6-H and 7-H), 1.65 (s, 3H, 27-CH_3_), 1.63–1.58 (m, 1H, 9-H), 1.54 (s, 3H, 26-CH_3_), 1.37 (s, 3H, 30-CH_3_), 1.35–1.08 (m, 3H, 15-H, 6-H and 7-H), 0.98 (s, 3H, 19-CH_3_), 0.92 (s, 3H, 18-CH_3_), 0.83 (s, 3H, 28-CH_3_). ^13^C NMR (100 MHz, CDCl_3_): δ = 174.01, 171.86, 171.32, 171.07, 149.87, 137.19, 133.93, 131.83, 129.96, 129.89, 129.65, 124.48, 75.82, 69.50, 67.79, 56.81, 56.39, 56.06, 55.64, 54.84, 50.56, 50.11, 47.42, 46.50, 45.37, 43.02, 40.86, 40.45, 39.05, 38.25, 37.17, 36.34, 33.94, 32.33, 31.17, 30.42, 29.75, 29.55, 28.61, 27.12, 25.71, 24.13, 22.36, 22.03, 19.37, 19.13, 17.02. HRMS-ESI *m*/*z*: calcd for C_47_H_68_N_2_O_8_ [*M*+H]^+^: 789.5048, found: 789.5037.

benzyl(Z)-2-((3R,4S,5S,8S,9S,10S,11R,13R,14S,16S)-3-((L-alanyl-L-alanyl)oxy)-16-acetoxy-11-hydroxy-4,8,10,14-tetramethylhexadecahydro-17H-cyclopenta[a]phenanthren-17-ylidene)-6-methylhept-5-enoate (**10**).

Referring to the synthesis of compound **7**, Intermediate **I** (424.8 mg, 0.7 mmol) was reacted with N-Boc-L-alanyl-L-alanine (244.3 mg, 1.0 mmol) with stirring at room temperature for 6 h. At the end of the reaction, it was diluted with ethyl acetate; washed twice each with 5% HCl solution, deionized water, and saturated sodium chloride solution; and then dried with anhydrous Na_2_SO_4_ and concentrated under reduced pressure. The dried intermediate was dissolved in anhydrous dichloromethane (20 mL), and an excess of TFA was added. The reaction was then carried out at room temperature with stirring for 1 h. At the end of the reaction, dichloromethane was added to dilute it. The mixture was washed with saturated sodium bicarbonate solution, deionized water, and saturated sodium chloride solution in turn; dried with anhydrous Na_2_SO_4_ and concentrated under reduced pressure; and then the residue was purified by silica gel column chromatography, using petroleum ether-ethyl acetate (50:1~10:1) as the eluent, to obtain compound **10** (461.3 mg; yield 79.1%). [α${]}_{D}^{20}$= −3.0 (c = 0.05, MeOH: CHCl_3_ = 3:2). m.p. 70.9–75.1 °C. R_f_ = 0.1 (dichloromethane/methanol = 20:1). ^1^H NMR (400 MHz, CDCl_3_): δ = 8.21 (s, 2H, -NH_2_), 7.78 (s, 1H, -NHCO-), 7.37–7.31 (m, 5H, Ar-H), 5.90 (dd, *J* = 41.0, 7.9 Hz, 1H, 16-H), 5.18 (d, *J* = 12.1 Hz, 1H, -CHAr), 4.99–4.82 (m, 2H, 3-H, -CHAr), 4.65–4.43 (m, 1H, -NCHCO-), 4.28 (s, 1H, 11-H), 4.19 (s, 1H, -NCHCO-), 3.00 (d, *J* = 12.7 Hz, 1H, 13-H), 2.41 (s, 2H, 2×22-H), 2.35–2.23 (m, 1H, 12-H), 2.24–1.94 (m, 5H, 15-H, 2×23-H, 1-H and 5-H), 1.91 (s, 3H, -OCOCH_3_), 1.45 (s, 3H, 27-CH_3_), 1.41 (s, 3H, 26-CH_3_), 1.30 (s, 3H, 30-CH_3_), 1.24 (s, 6H, 2×-CH_3_), 0.93 (s, 3H, 19-CH_3_), 0.88 (s, 3H, 18-CH_3_), 0.79 (d, *J* = 6.0 Hz, 3H, 28-CH_3_). ^13^C NMR (100 MHz, CDCl_3_): δ = 171.25, 169.45, 166.71, 134.75, 131.49, 131.30, 129.90, 128.79, 127.83, 127.57, 127.53, 127.44, 122.05, 109.84, 109.18, 88.11, 73.17, 65.38, 64.56, 47.62, 44.14, 42.92, 38.46, 37.93, 36.36, 35.57, 34.24, 30.91, 29.56, 28.68, 26.02, 24.64, 24.29, 24.21, 22.74, 22.16, 21.67, 21.30, 19.92, 18.17, 16.69, 16.66, 14.55, 12.71. HRMS-ESI *m*/*z*: calcd for C_44_H_64_N_2_O_8_ [*M*+Na]^+^: 771.4555, found: 771.4537.

3-((3-(((3R,4S,5S,8S,9S,10S,11R,13R,14S,16S,Z)-16-acetoxy-17-(1-(benzyloxy)-6-methyl-1-oxohept-5-en-2-ylidene)-11-hydroxy-4,8,10,14-tetramethylhexadecahydro-1H-cyclopenta[a]phenanthren-3-yl)oxy)-3-oxopropyl)disulfaneyl)propanoic acid (**11**).

Referring to the synthesis of compound 7, Intermediate I (424.8 mg, 0.7 mmol) was reacted with 3,3′-dithiodipropionic acid (210.3 mg, 1.0 mmol). The residue was purified by silica gel column chromatography, using petroleum ether-ethyl acetate (20:1~8:1) as the eluent, to obtain compound 11 (487.2 mg; yield 87.1%). R_f_ = 0.4 (dichloromethane/methanol = 20:1). ^1^H NMR (400 MHz, CDCl_3_): δ = 7.41–7.27 (m, 5H, Ar-H), 5.86 (d, *J* = 8.4 Hz, 1H, 16-H), 5.20 (d, *J* = 12.2 Hz, 1H, -CHAr), 5.04 (t, *J* = 7.1 Hz, 1H, 24-H), 4.98–4.85 (m, 2H, 3-H, -CHAr), 4.36 (s, 1H, 11-H), 3.00 (s, 1H, 13-H), 2.93 (q, *J* = 7.4, 6.5 Hz, 4H, 2×-SCH_2_-), 2.86–2.60 (m, 4H, 2×-COCH_2_-), 2.51–2.34 (m, 2H, 2×22-H), 2.28 (d, *J* = 13.4 Hz, 1H, 12-H), 2.18–1.96 (m, 5H, 15-H, 2×23-H, 1-H and 5-H), 1.92 (s, 3H, -OCOCH_3_), 1.62 (s, 3H, 27-CH_3_), 1.51 (s, 3H, 26-CH_3_), 1.35 (s, 3H, 30-CH_3_), 0.97 (s, 3H, 19-CH_3_), 0.90 (s, 3H, 18-CH_3_), 0.81 (d, *J* = 6.7 Hz, 3H, 28-CH_3_). ^13^C NMR (100 MHz, CDCl_3_): δ = 171.30, 170.53, 169.91, 167.73, 148.25, 135.71, 132.58, 132.29, 130.92, 130.46, 128.84, 128.55, 128.48, 128.26, 123.00, 74.88, 74.38, 68.46, 66.42, 65.58, 49.17, 48.65, 43.87, 39.40, 37.39, 36.72, 35.09, 34.63, 33.78, 33.14, 32.12, 30.56, 29.01, 28.34, 27.22, 25.70, 23.93, 23.00, 20.93, 19.18, 17.82, 17.71, 15.65, 13.70. HRMS-ESI *m*/*z*: calcd for C_44_H_62_O_9_S_2_ [*M*+Na]^+^: 821.3727, found: 821.3710.

### 3.8. Biological Activity Evaluation

(1) Inhibition Rate. In this study, FA-resistant MRSA (MIC_90_ = 32 μg/mL) was used [15,56]. An appropriate number of MRSA (ATCC 43300) colonies in the logarithmic growth phase were cultured in a sterile liquid medium at 37 °C for 6–8 h until the optical density at 600 nm (OD600) reached approximately 0.5. The bacterial suspension was diluted to a final concentration of 1 × 10^6^ CFU/mL. The test compound was prepared as a stock solution at a concentration of 1 mg/mL. Then, 500 μL of the bacterial suspension was mixed with 500 μL of the compound solution, resulting in a final compound concentration of 500 μg/mL. Following a 24 h incubation period, 100 μL of the culture from the shaking tube was spread onto the agar plates using the spread-plate method. The plates were incubated at 37 °C for 24 h, and then the colony-forming units (CFUs) were calculated to determine the inhibition rate.

(2) Determination of MIC Value. A serial dilution of the compound was prepared with concentrations of 256, 128, 64, 32, 16, 8, 4, 2, 1, and 0.5 μg/mL. Positive and negative control conditions were established, with each well containing a bacterial inoculum at a concentration of 2.5 × 106 CFU/mL. The 96-well plates were incubated at 37 °C for 24 h. A microplate reader measured the absorbance at 600 nm (OD600) for each well. The value of minimum concentrations required to inhibit 90% of bacterial growth (MIC_90_) for the FA derivatives was determined.

### 3.9. Cell Cytotoxicity Assay

Regarding the ISO 10993-5:2009(E) standard and previously published study [32,57], the human embryonic kidney cells (293T) in the logarithmic growth phase were seeded into a 96-well plate with a density of 5 × 10^3^ cells per well and incubated overnight. FA and compounds **4** or **10** (40, 20, 10, 5, 1, and 0.5 μM) were then added, and the incubation was continued for 24 h. Methylthiazolyldiphenyl-tetrazolium bromide solution was added to each well and incubated for 4 h, followed by removing the medium and adding DMSO (150 μL) to dissolve the formazan. The absorbance was measured under 490 nm, and the cell viability was calculated according to the formula below:

(1) $$\mathrm{Cell}\;\mathrm{Viability}\left(\%\right)=\frac{A_{\mathrm{Sample}}-A_{\mathrm{Blank}}}{A_{\mathrm{Control}}-A_{\mathrm{Blank}}}\times 100\%$$

## 4. Conclusions

In this study, we built a stepwise procedure to discover novel FA derivatives. First, the molecular modeling of FA and wildtype or mutant EF-G enabled the identification of key residues in EF-G involved in the stability of the target and the interactions with FA and preliminarily elucidated the molecular mechanism of drug resistance in MRSA. Then, we employed an AI-based molecular generator and screening models to quickly identify promising hits from massive-scale compounds based on exploring a broader chemical space compared to the experience-based design. Finally, the wet experiments enabled us to discover two novel FA derivatives with high antibacterial activity superior to that of FA. Future studies should focus on the optimization of the design and screening models from the view of drug-likeness and generate novel compounds with good pharmacokinetics and efficacy in the in vivo models.

## Figures and Tables

**Table 1:** Docking scores and antimicrobial activities of FA and 5 derivatives modified at 21-COOH of FA.

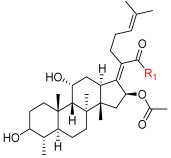					
NO.	R_1_	Chemprop	RTMScore	Inhibition Rate (%)	MIC_90_ (μg/mL)
FA (**1**)	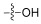	0.860	20.3	100	32
**2**	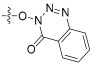	0.990	27.5	2.16	-
**3**	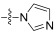	0.990	24.9	1.08	-
**4**	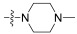	0.989	25.3	100	16
**5**	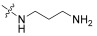	0.977	26.8	100	32
**6**	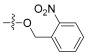	0.992	30.4	2.16	-

**Table 2:** Docking scores and antimicrobial activities of FA and 5 derivatives modified at 3-OH of FA.

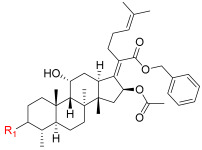					
NO.	R_1_	Chemprop	RTMScore	Inhibition Rate (%)	MIC_90_ (μg/mL)
FA (**1**)	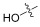	0.860	20.3	100	32
**7**	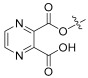	0.987	32.1	100	32
**8**	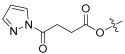	0.993	29.9	93.13	128
**9**	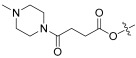	0.991	30.3	99.98	32
**1** **0**	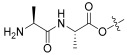	0.978	25.8	100	16
**1** **1**	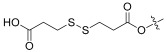	0.979	28.7	99.98	32

## Data Availability

The data have been fully provided.

## Supplementary Materials

The following supporting information can be downloaded at https://www.mdpi.com/article/10.3390/molecules30091983/s1: Table S1. Absorbance data for different concentrations of the compounds at 600 nm. Figure S1. The 2D plots of wildtype (A), MUT-1 (B), MUT-2 (C), and MUT-3 (D) interacted with FA analyzed by Ligplot+ (Version v.2.2) [58]. Figure S2. Generated examples based on Scaffold Generator-Decorator. Figure S3. The 2D plots of EF-G interacting with active compounds **4**, **5**, and **7**–**11**. Figure S4. Absorption–concentration plots of the compounds measured at 600 nm. Figures S5–S14. ^1^H NMR (400 MHz, CDCl_3_), ^13^C NMR (100 MHz, CDCl_3_), and HRMS (ESI) spectra of compounds **2**–**11**.
