# Supplementary material for: Multi-Modal Design, Synthesis, and Biological Evaluation of Novel Fusidic Acid Derivatives
Source: Molecules. 2025 Apr 29;30(9):1983. doi: 10.3390/molecules30091983 (PMC12073777; doi:10.3390/molecules30091983)
Supplement: Supplementary file 1 [file molecules-30-01983-s001.zip › molecules-3595315-supplementary.pdf]

Supporting Information

Multi-modal design, synthesis, and biological evaluation of novel fusidic acid derivatives.

Luqi Wang [†], Zhiyuan Geng [†], Yuhang Liu, Linhui Cao, Yao Liu, Hourui Zhang, Yi Bi* and Jing Lu*

School of Pharmacy, Key Laboratory of Molecular Pharmacology and Drug Evaluation (Yantai University), Ministry of Education, Collaborative Innovation Center of Advanced Drug Delivery System and Biotech Drugs in Universities of Shandong, Yantai University, Yantai 264005, China

[†] Authors contributed equally to this manuscript.

* Correspondence : Jing Lu, lujing_ytu@126.com; Yi Bi, beeyee_413@163.com; phone/fax: 86-0535-6706066

Contents:

1. Tables S1 ……………………………………………………………………….S2

2. Figure S1–S4 ...………………..……………………………………..……….S3–6

3. ^{1}H NMR, ^{13}C NMR, and HRMS spectra of target compounds (**2**–**11**) ….……S7–24

Table S1. Absorbance data for different concentrations of the compounds at 600 nm.

concentration（μg/mL）	256	128	64	32	16	8	4	2	1	0.5	positive control	negative control
	0.016	0.019	0.022	0.023	0.589	0.612	0.608	0.611	0.613	0.621	0.633	0.014
FA(**1**)	0.015	0.017	0.021	0.022	0.587	0.615	0.616	0.617	0.621	0.624	0.634	0.015
	0.017	0.018	0.023	0.027	0.561	0.613	0.611	0.613	0.615	0.625	0.637	0.013
	0.015	0.016	0.021	0.023	0.024	0.587	0.594	0.609	0.614	0.622	0.629	0.012
**4**	0.014	0.017	0.02	0.021	0.023	0.589	0.593	0.608	0.612	0.617	0.623	0.013
	0.016	0.018	0.023	0.022	0.027	0.592	0.599	0.606	0.615	0.618	0.625	0.014
	0.016	0.021	0.022	0.027	0.591	0.602	0.607	0.613	0.616	0.612	0.619	0.015
**5**	0.013	0.019	0.021	0.026	0.593	0.606	0.609	0.614	0.617	0.623	0.639	0.012
	0.018	0.022	0.022	0.026	0.59	0.601	0.608	0.615	0.615	0.617	0.621	0.016
	0.013	0.018	0.015	0.021	0.579	0.602	0.611	0.614	0.612	0.622	0.621	0.015
**7**	0.014	0.017	0.019	0.022	0.578	0.601	0.612	0.613	0.616	0.624	0.623	0.017
	0.016	0.018	0.016	0.023	0.589	0.604	0.614	0.616	0.621	0.623	0.625	0.016
	0.016	0.021	0.569	0.575	0.573	0.589	0.601	0.605	0.614	0.621	0.635	0.016
**8**	0.014	0.022	0.571	0.578	0.581	0.591	0.603	0.607	0.615	0.624	0.637	0.015
	0.013	0.019	0.568	0.573	0.577	0.586	0.602	0.611	0.615	0.622	0.636	0.016
	0.014	0.015	0.018	0.022	0.602	0.605	0.611	0.621	0.618	0.62	0.627	0.014
**9**	0.016	0.015	0.017	0.024	0.604	0.609	0.612	0.623	0.621	0.624	0.629	0.012
	0.013	0.017	0.019	0.028	0.609	0.611	0.612	0.622	0.625	0.627	0.631	0.015
	0.018	0.021	0.025	0.023	0.031	0.589	0.598	0.602	0.612	0.619	0.626	0.015
**10**	0.015	0.022	0.026	0.024	0.033	0.587	0.598	0.603	0.615	0.618	0.623	0.014
	0.021	0.019	0.023	0.025	0.037	0.592	0.601	0.605	0.613	0.619	0.631	0.017
	0.015	0.016	0.018	0.021	0.587	0.601	0.602	0.611	0.609	0.618	0.632	0.013
**11**	0.017	0.018	0.021	0.023	0.589	0.603	0.607	0.614	0.619	0.624	0.633	0.014
	0.013	0.017	0.019	0.022	0.591	0.605	0.609	0.612	0.616	0.619	0.631	0.011

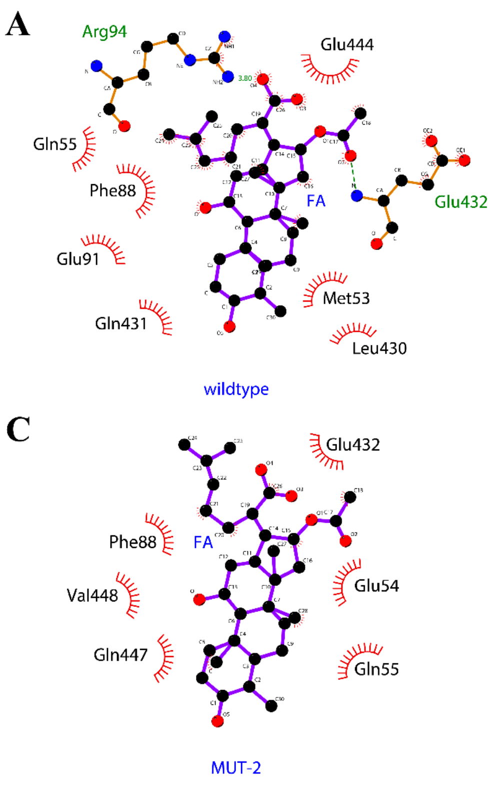

Figure S1. The 2D plots of wildtype (A), MUT-1 (B), MUT-2 (C), and MUT-3 (D) interacted with FA analyzed by Ligplot+ (Version v.2.2)[1].

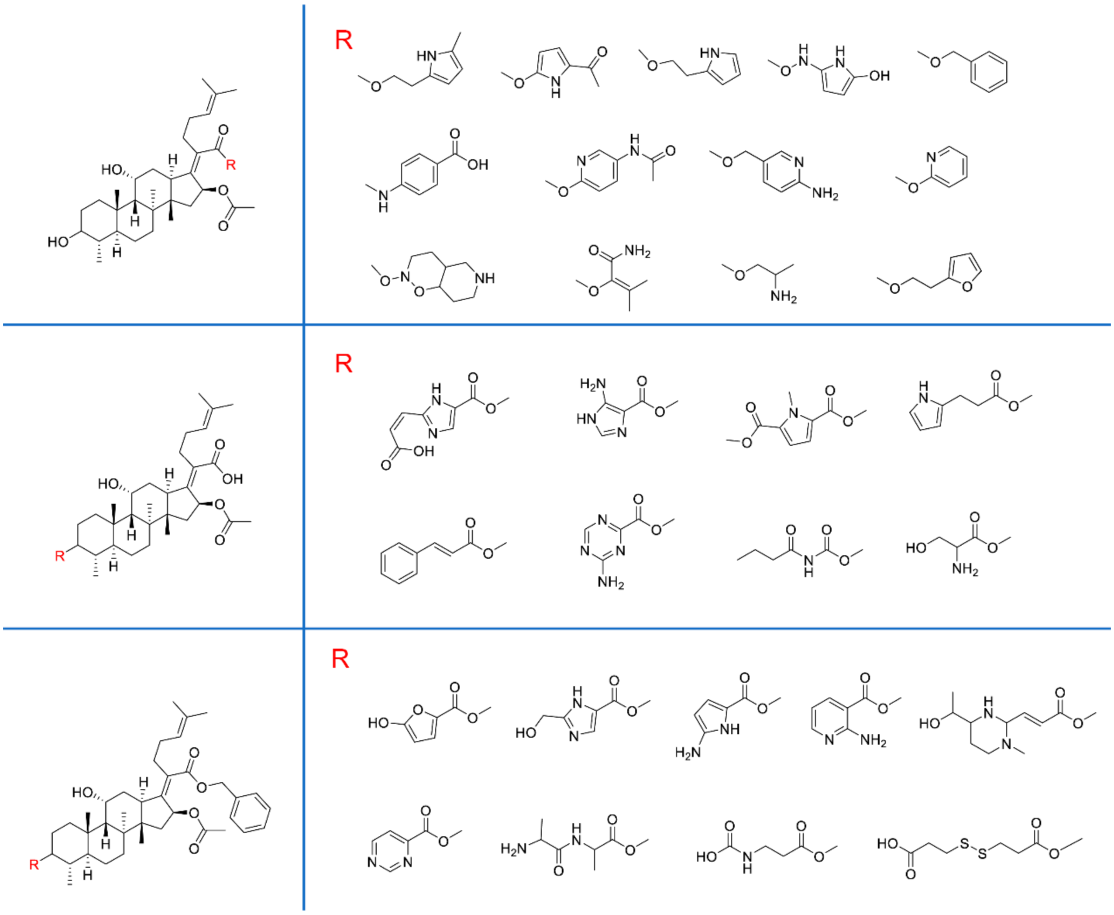

Figure S2. Generated examples based on Scaffold Generator-Decorator.

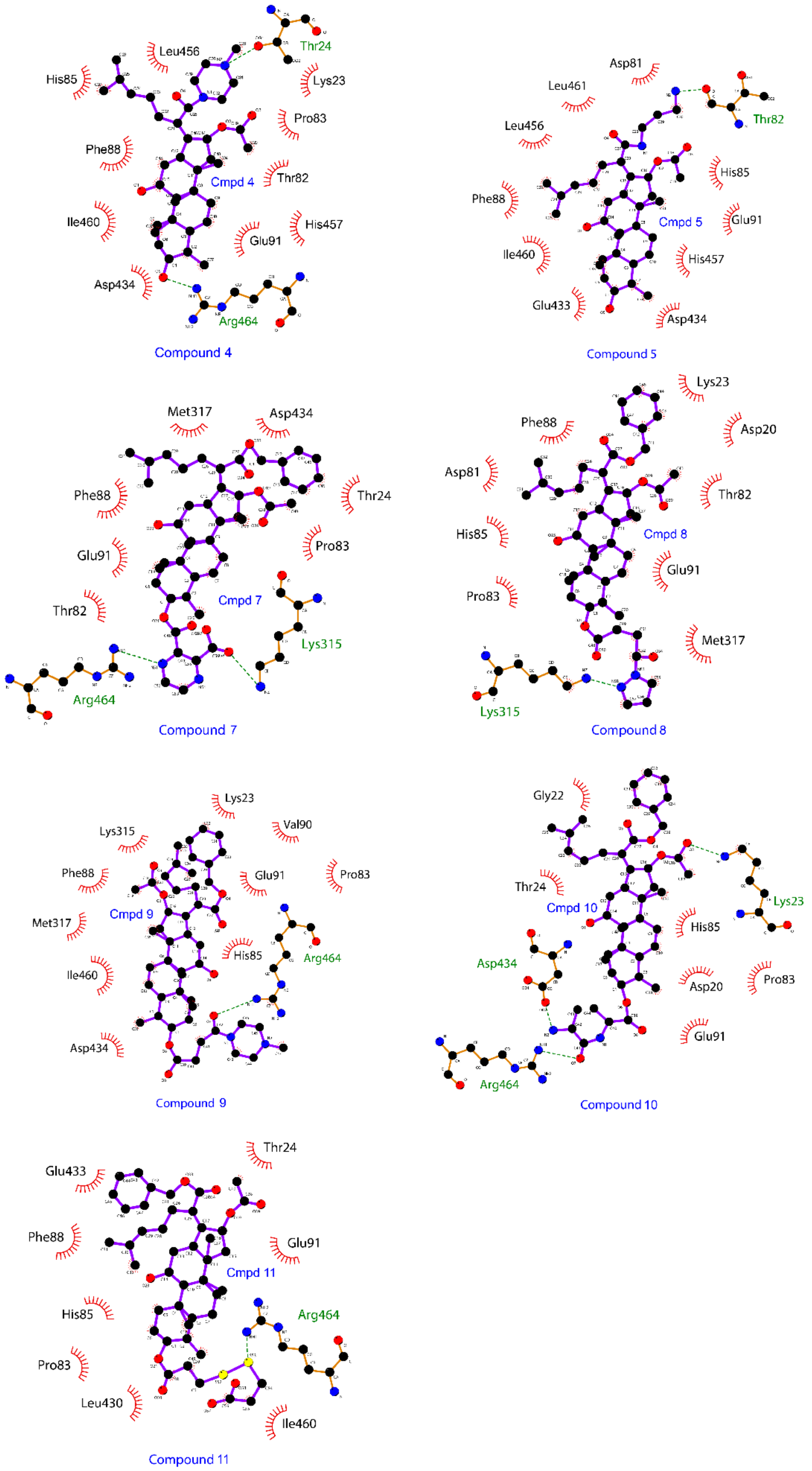

Figure S3. The 2D plots of EF-G interacting with active compounds **4**, **5**, and **7–11**. Cmpd means compound.

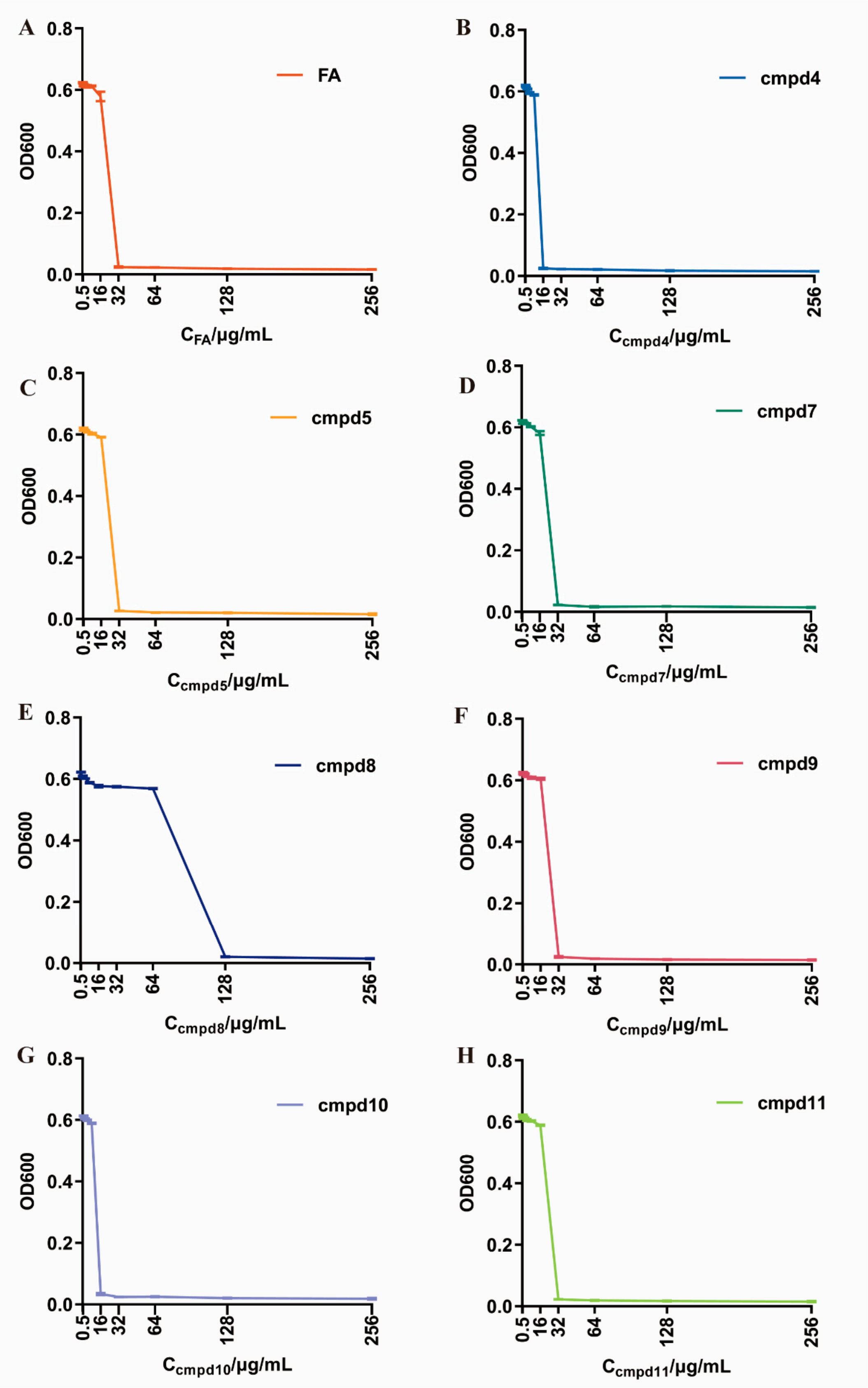

Figure S4. Absorption–concentration plots of the compounds measured at 600 nm.

^{1}H NMR (400 MHz, CDCl$_3$), ^{13}C NMR (100 MHz, CDCl$_3$), and HRMS (ESI) spectra of compounds 2–11.

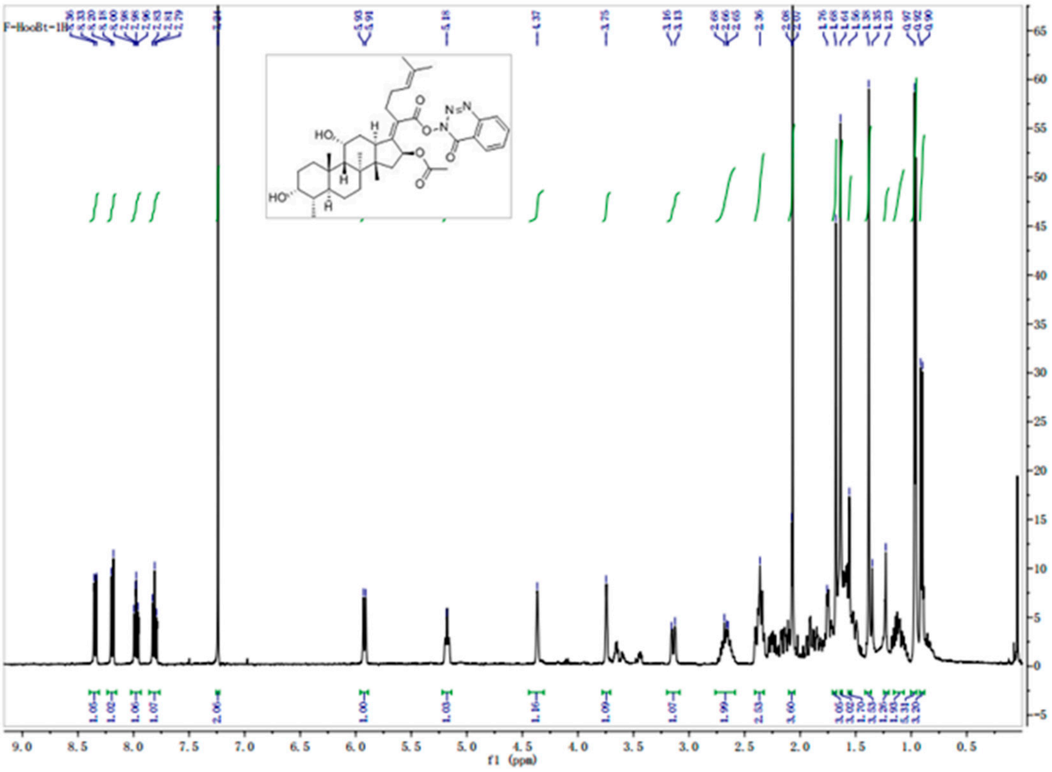

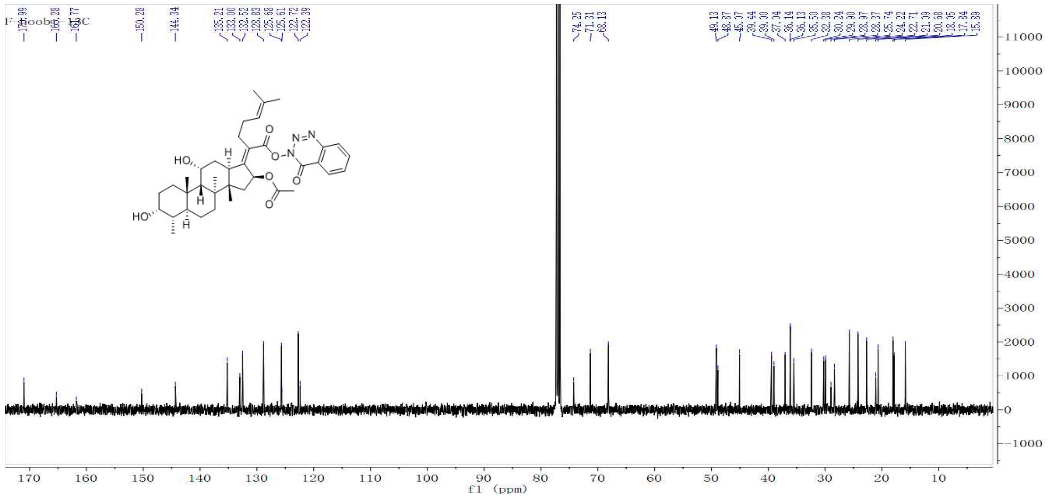

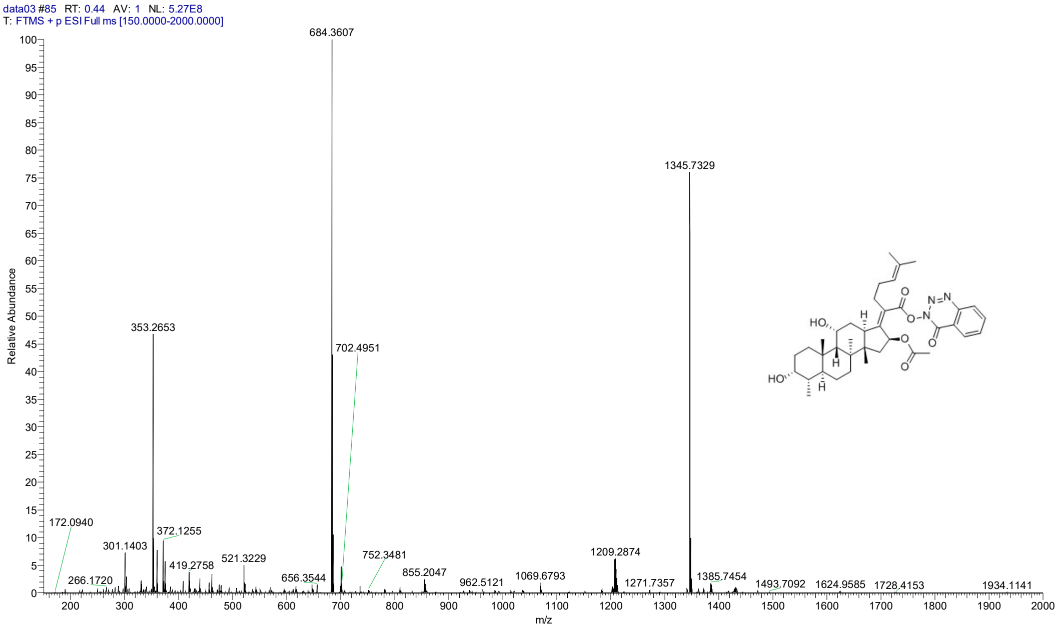

Figure S5. ^{1}H NMR (400 MHz, $CDCl_3$), ^{13}C NMR (100 MHz, $CDCl_3$), and HRMS (ESI) spectra of compound **2.**

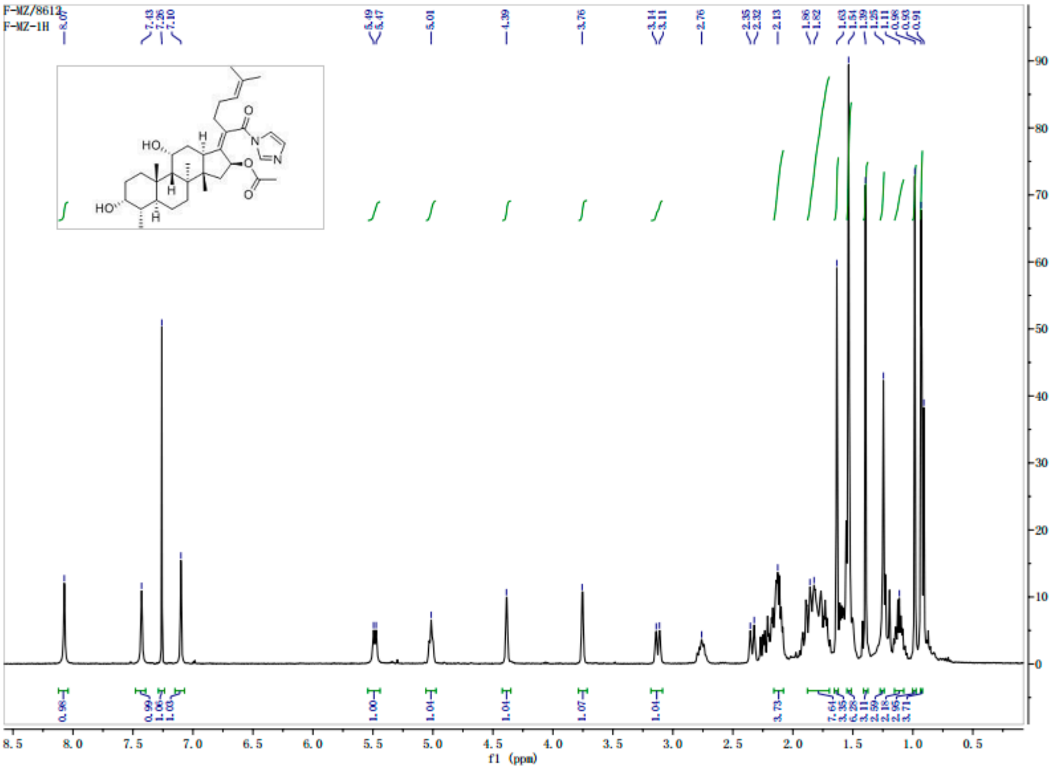

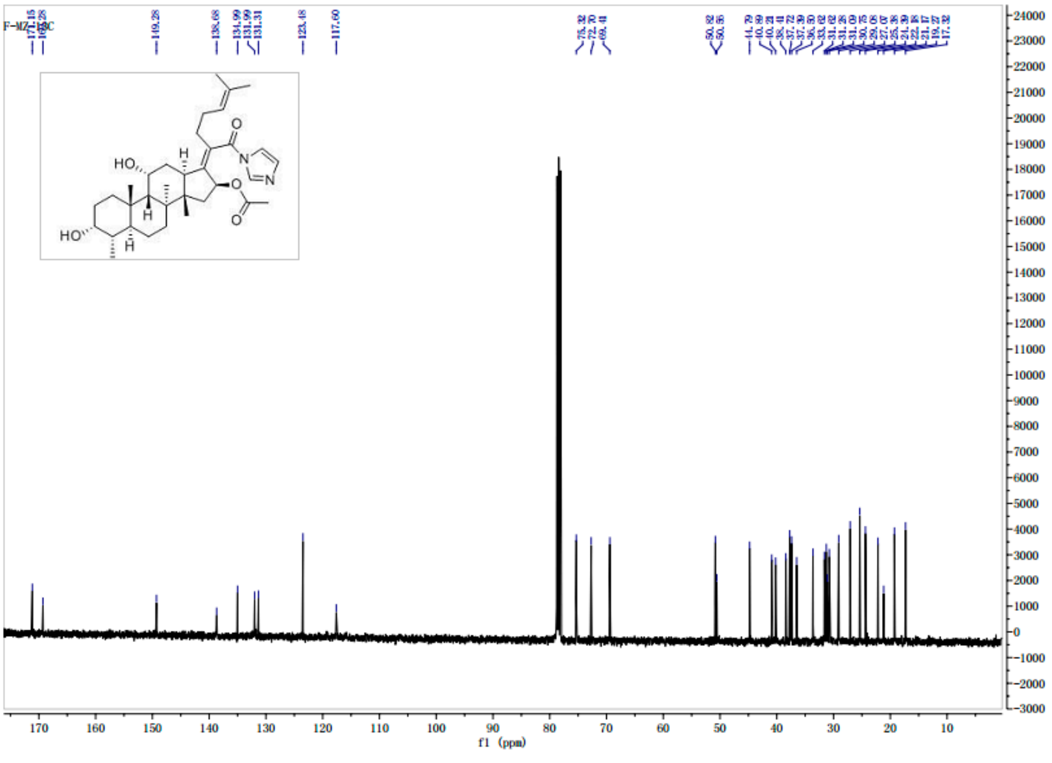

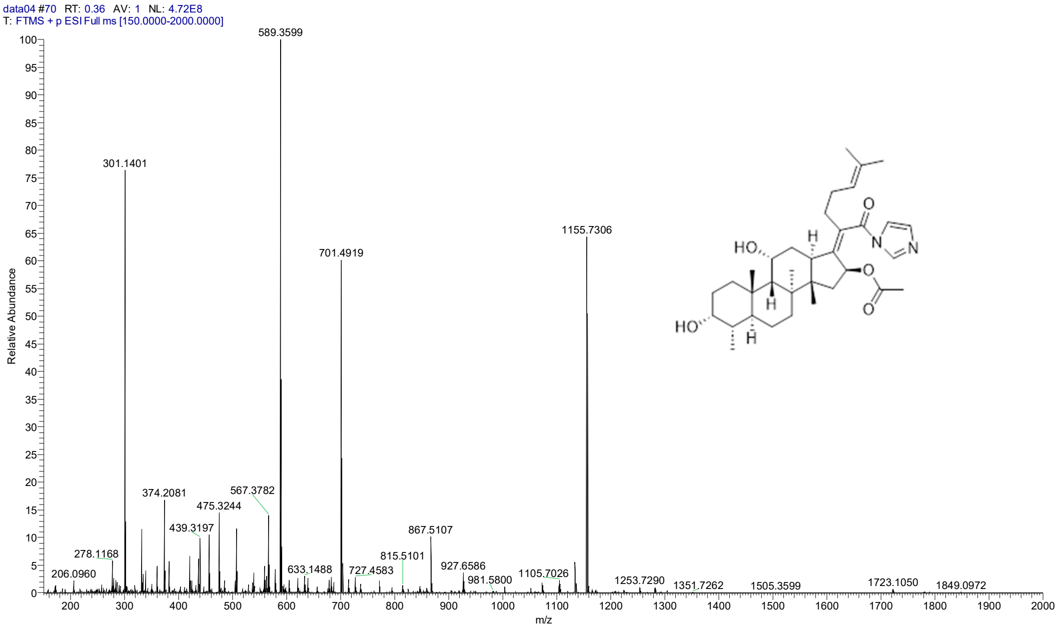

Figure S6. ^{1}H NMR (400 MHz, $CDCl_3$), ^{13}C NMR (100 MHz, $CDCl_3$), and HRMS (ESI) spectra of compound **3.**

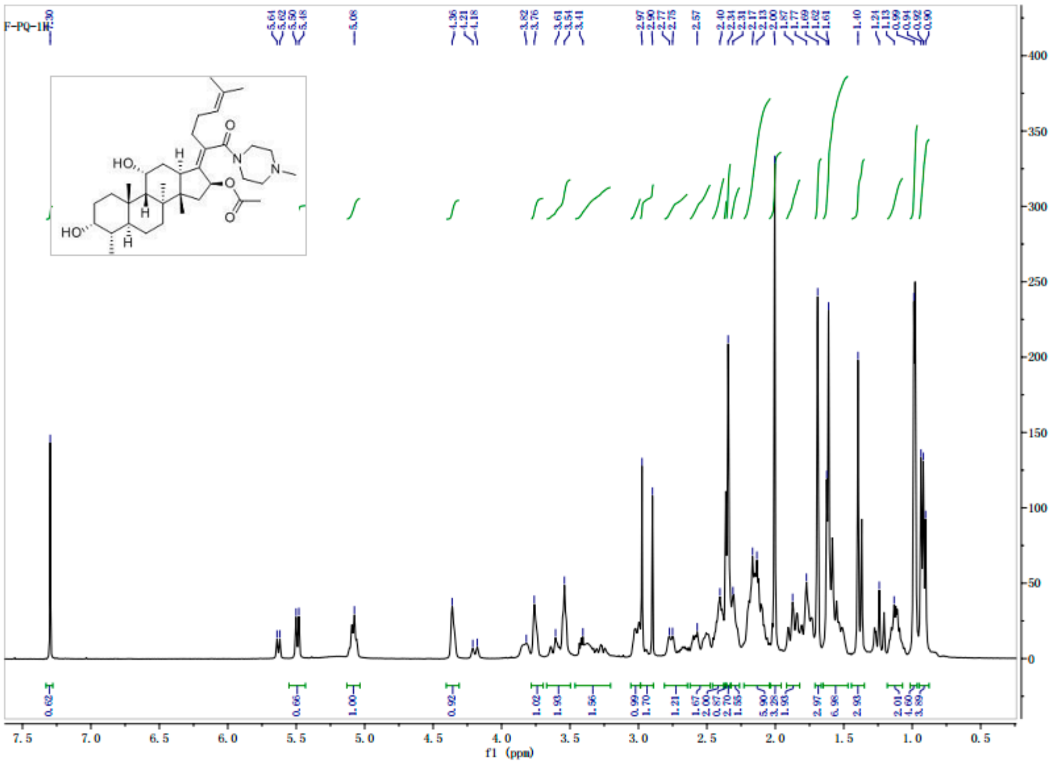
f1 (ppm)

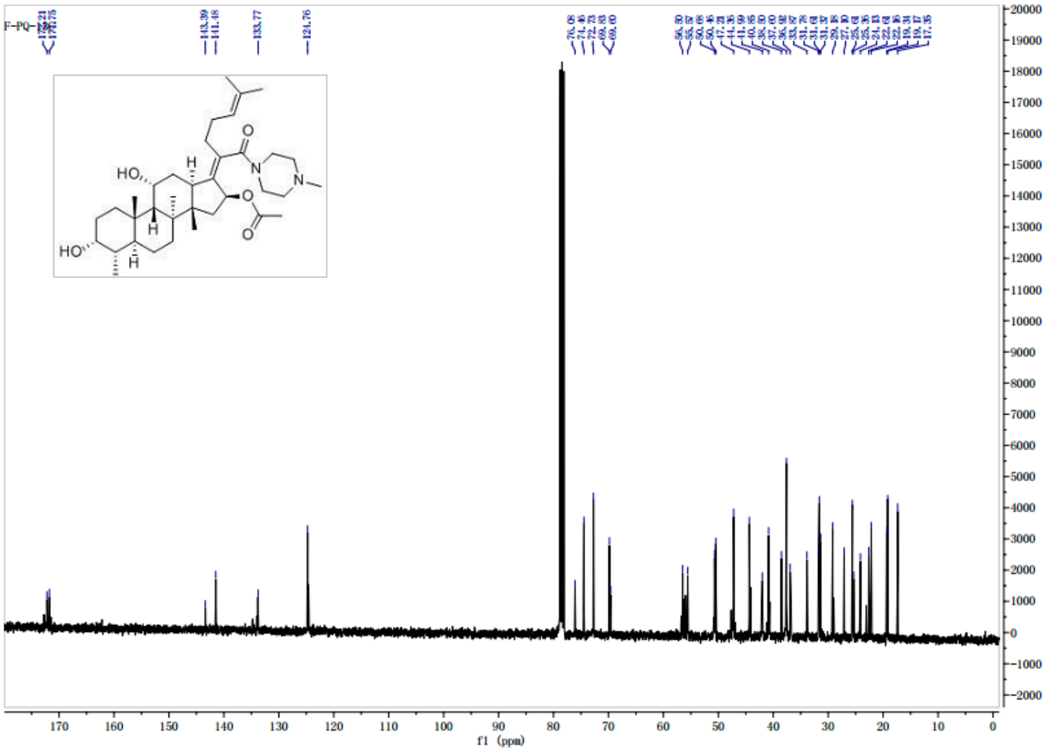
f1 (ppm)

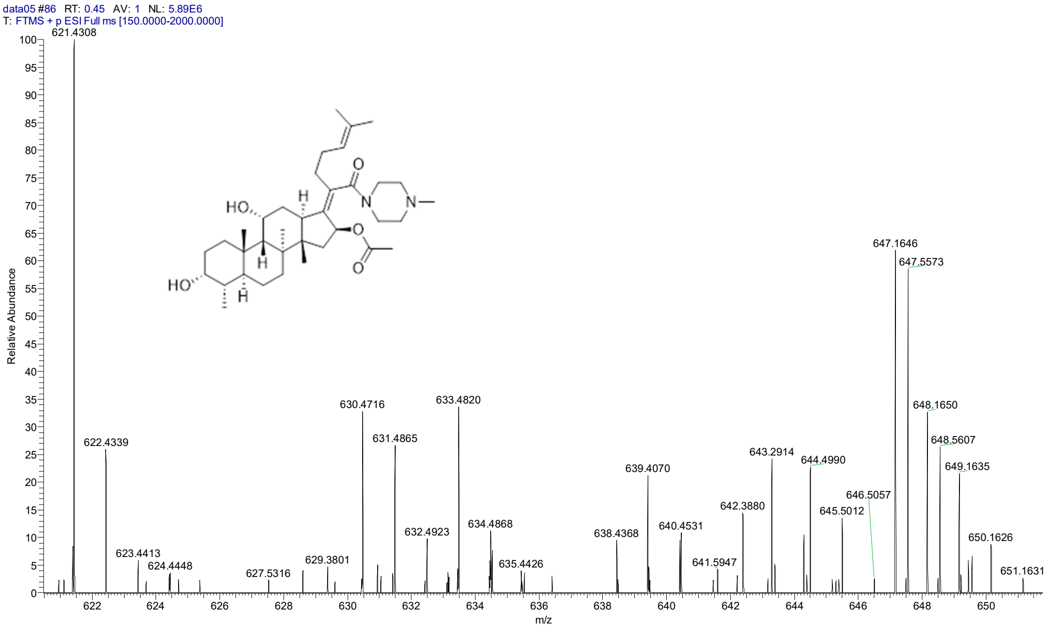

Figure S7. ^{1}H NMR (400 MHz, $CDCl_3$), ^{13}C NMR (100 MHz, $CDCl_3$), and HRMS (ESI) spectra of compound **4.**

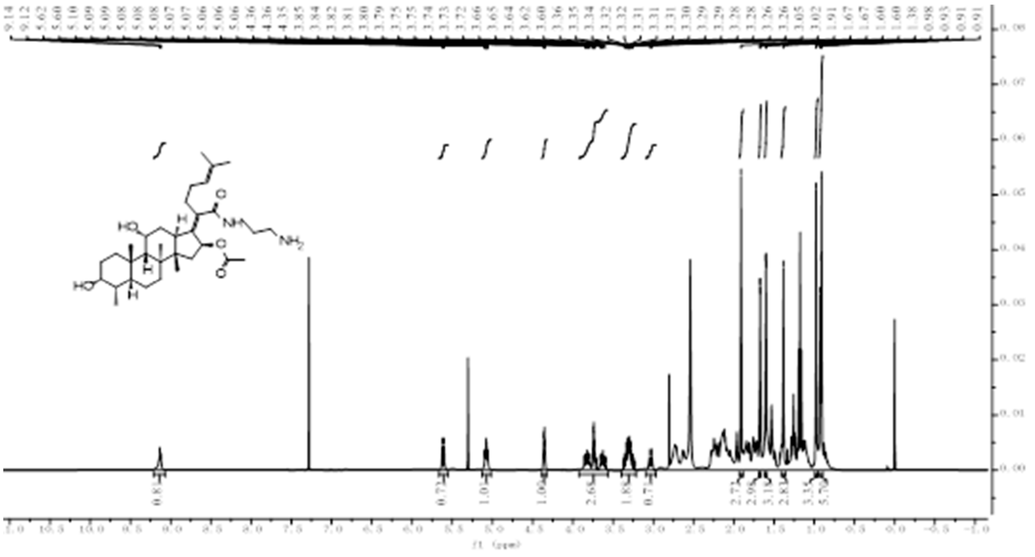
f1 (ppm)

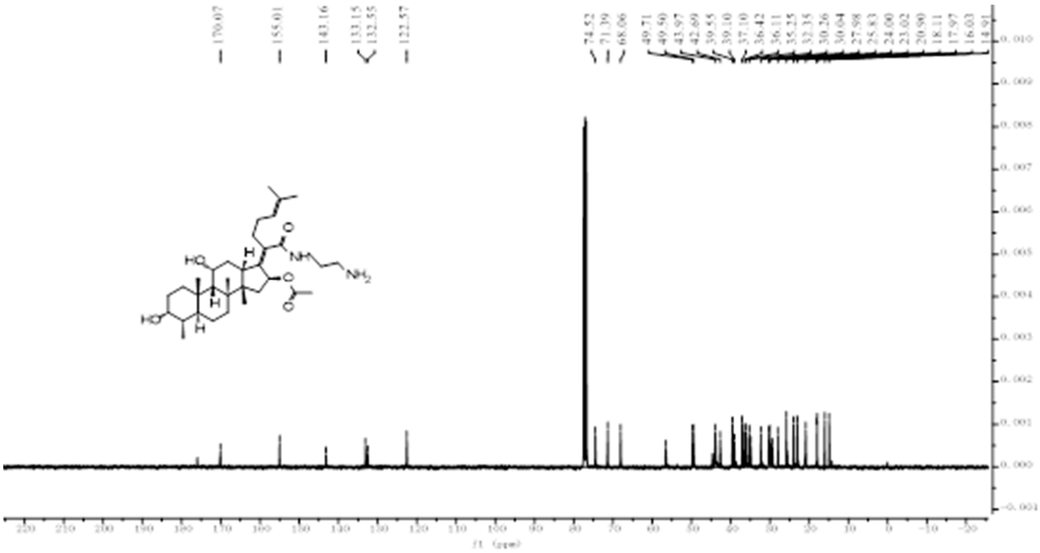
f1 (ppm)

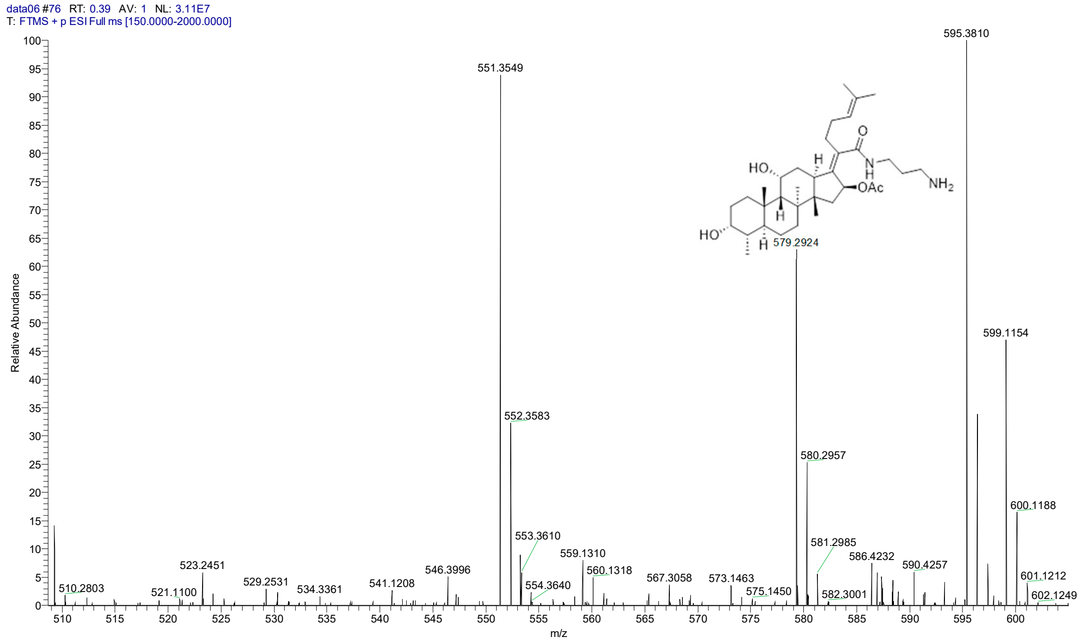

Figure S8. ^{1}H NMR (400 MHz, $CDCl_3$), ^{13}C NMR (100 MHz, $CDCl_3$), and HRMS (ESI) spectra of compound **5.**

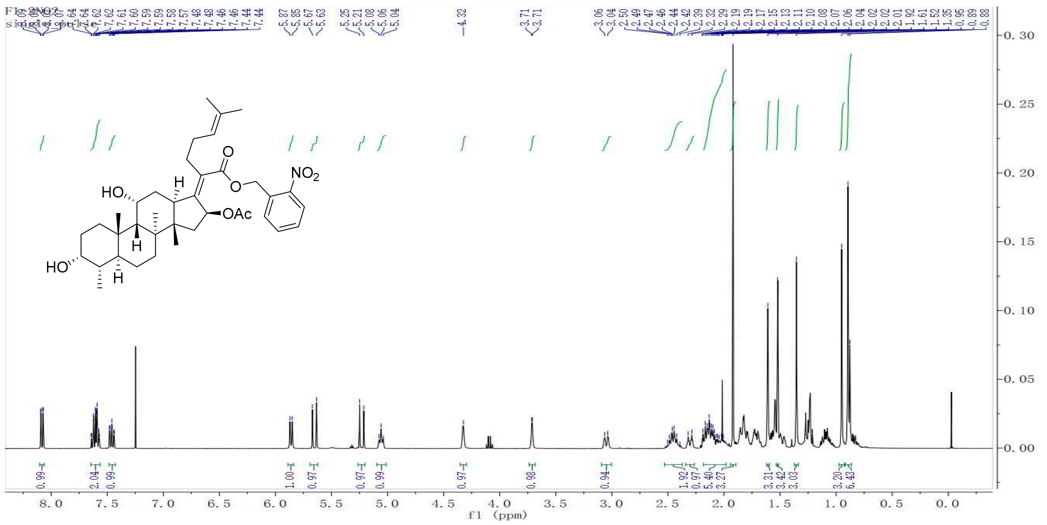

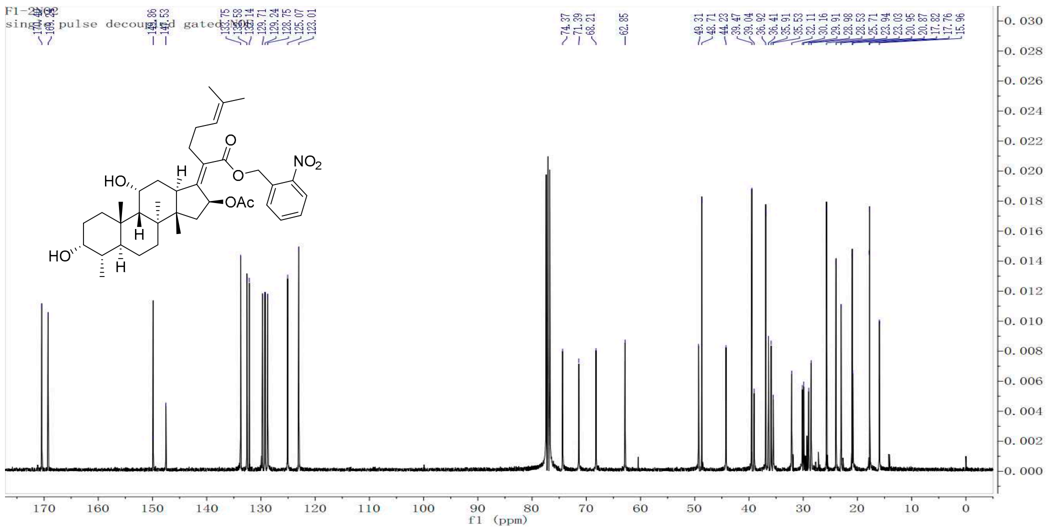

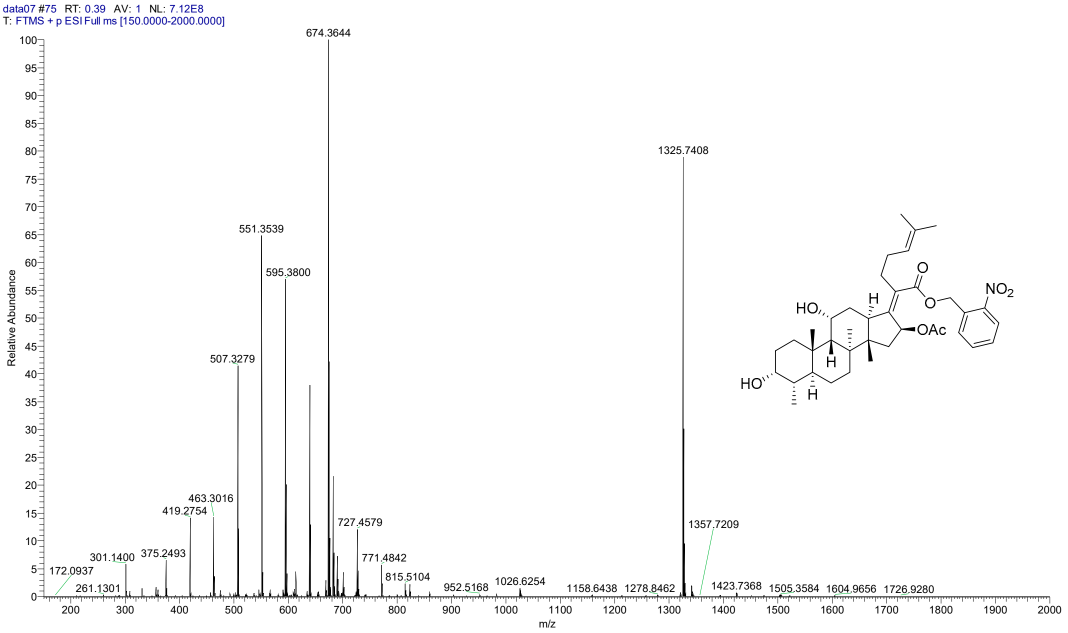

Figure S9. ^{1}H NMR (400 MHz, $CDCl_3$), ^{13}C NMR (100 MHz, $CDCl_3$), and HRMS (ESI) spectra of compound **6.**

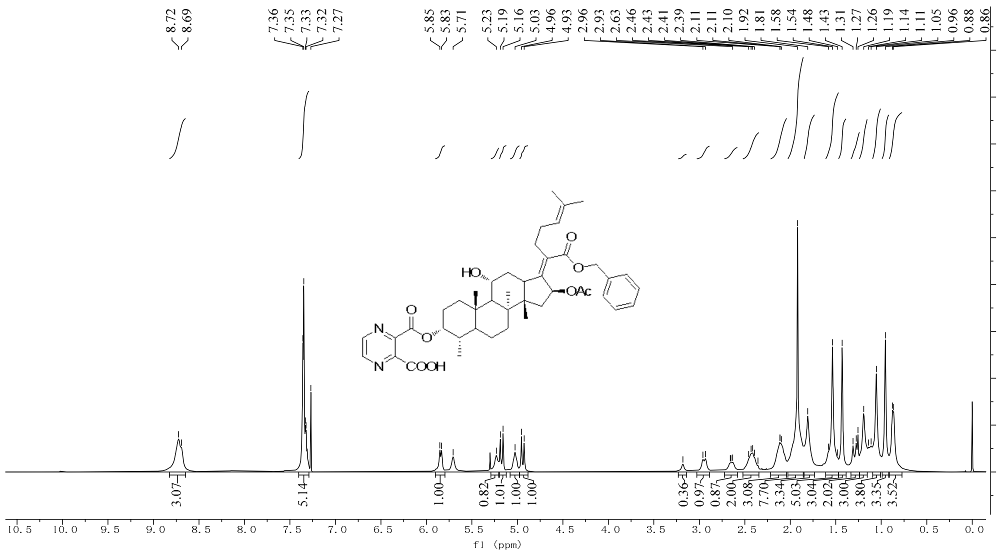
8.72
8.69
7.36
7.35
7.33
7.32
7.27
5.85
5.83
5.71
5.23
5.19
5.16
5.03
4.96
4.93
2.96
2.93
2.63
2.46
2.43
2.41
2.39
2.11
2.11
2.10
1.92
1.81
1.58
1.54
1.48
1.43
1.31
1.27
1.26
1.19
1.14
1.11
1.05
0.96
0.88
0.86
HO
OAc
N
COOH
f1 (ppm)

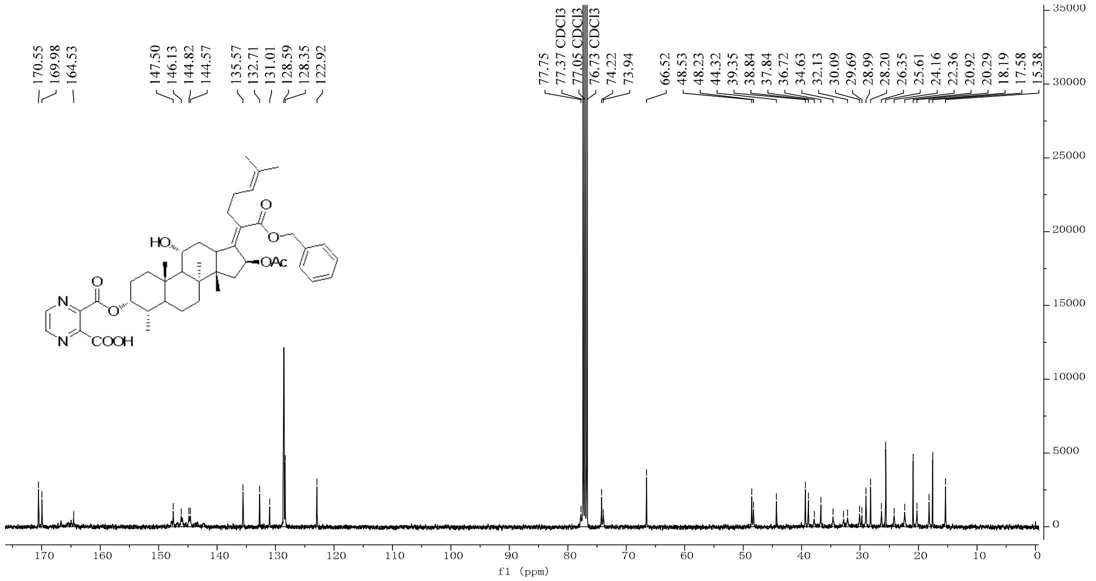
170.55
169.98
164.53
147.50
146.13
144.82
144.57
135.57
132.71
131.01
128.59
128.35
122.92
77.75
77.37 CDCl3
77.05 CDCl3
76.73 CDCl3
74.22
73.94
66.52
48.53
48.23
44.32
39.35
38.84
37.84
36.72
34.63
32.13
30.09
29.69
28.99
28.20
26.35
25.61
24.16
22.36
20.92
20.29
18.19
17.58
15.38
HO
OAc
N
COOH
f1 (ppm)

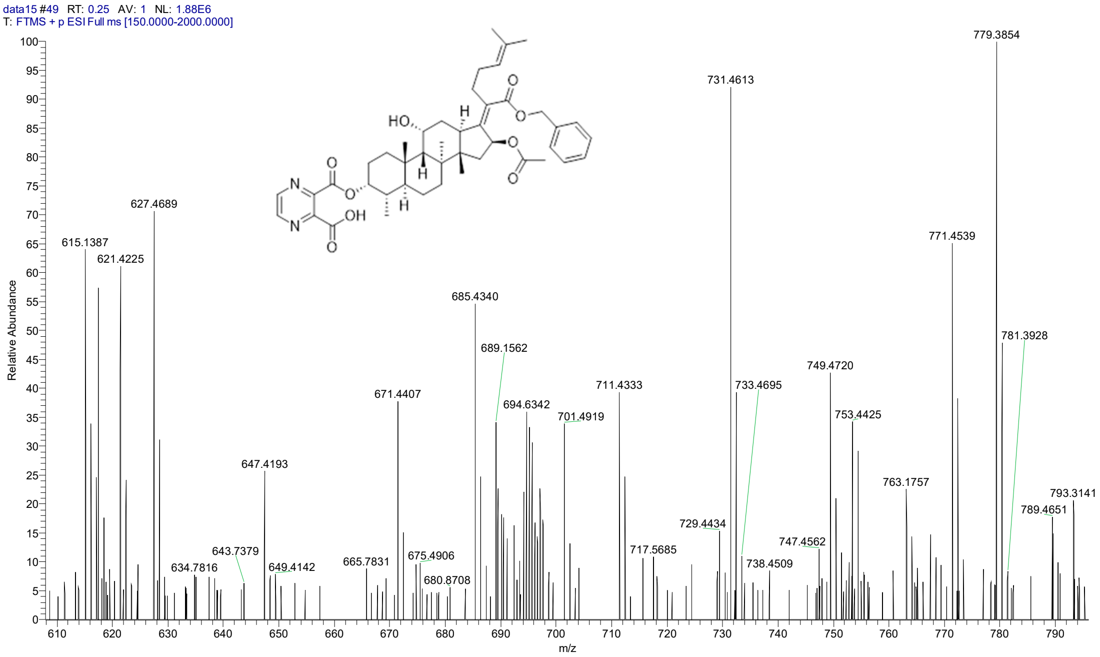

Figure S10. ^{1}H NMR (400 MHz, $CDCl_3$), ^{13}C NMR (100 MHz, $CDCl_3$), and HRMS (ESI) spectra of compound **7.**

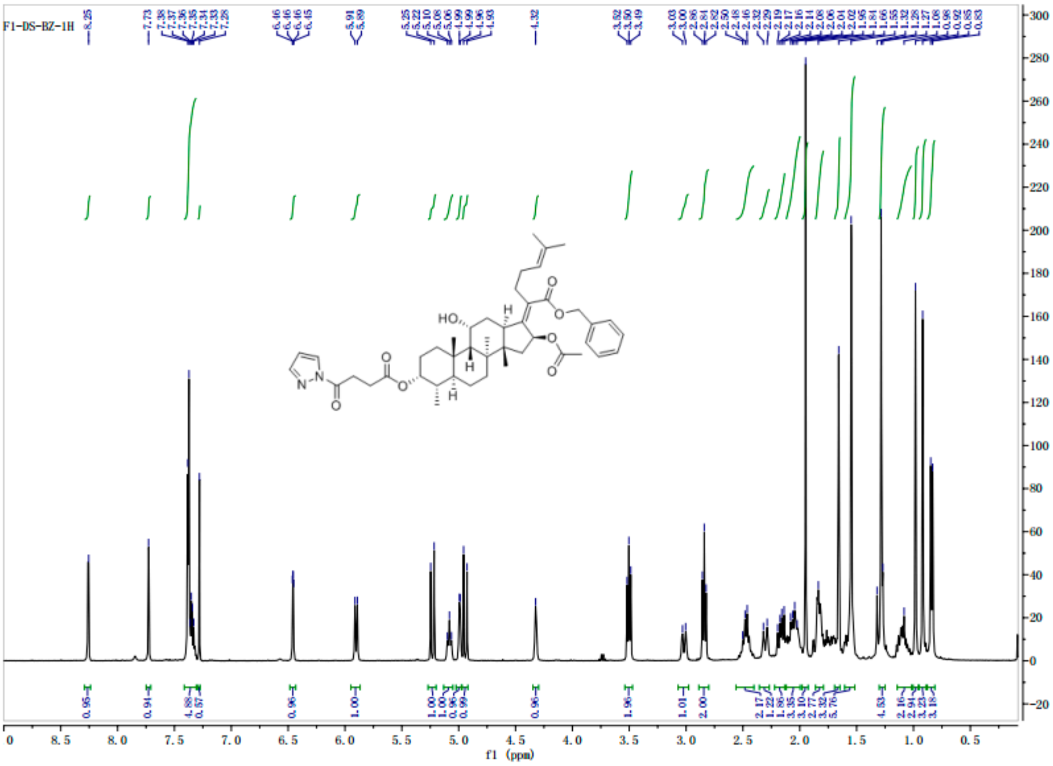
F1-DS-BZ-1H
f1 (ppm)

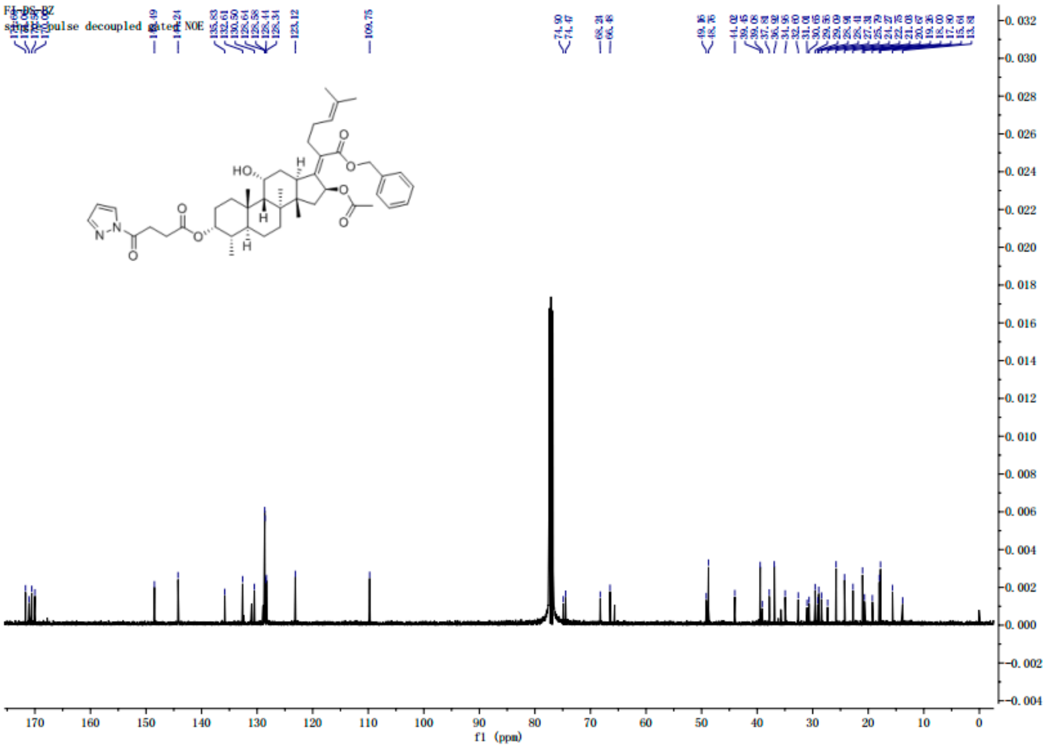
F1-DS-BZ
single pulse decoupled gated NOE
f1 (ppm)

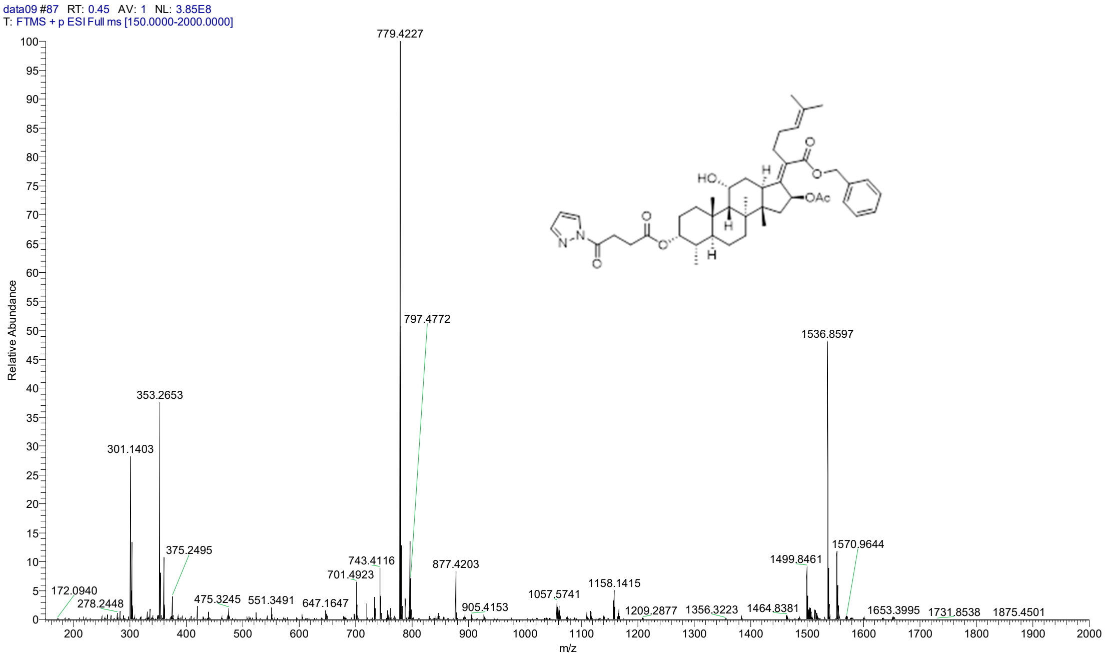

Figure S11. ^{1}H NMR (400 MHz, $CDCl_3$), ^{13}C NMR (100 MHz, $CDCl_3$), and HRMS (ESI) spectra of compound **8.**

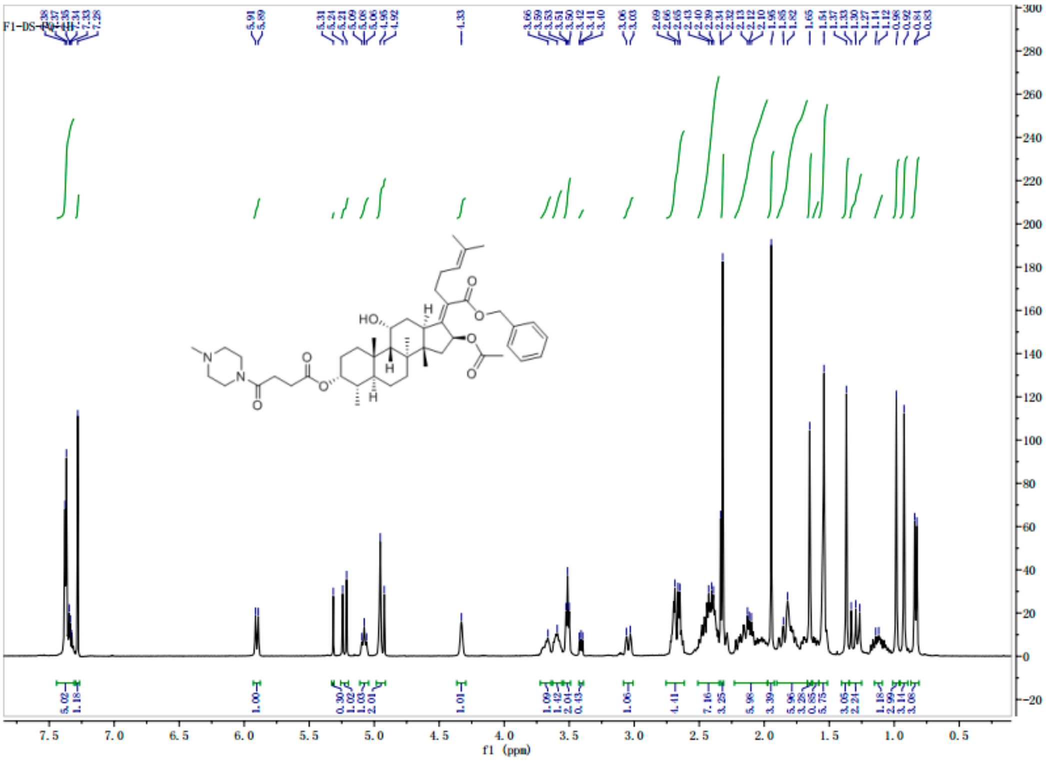
f1 (ppm)

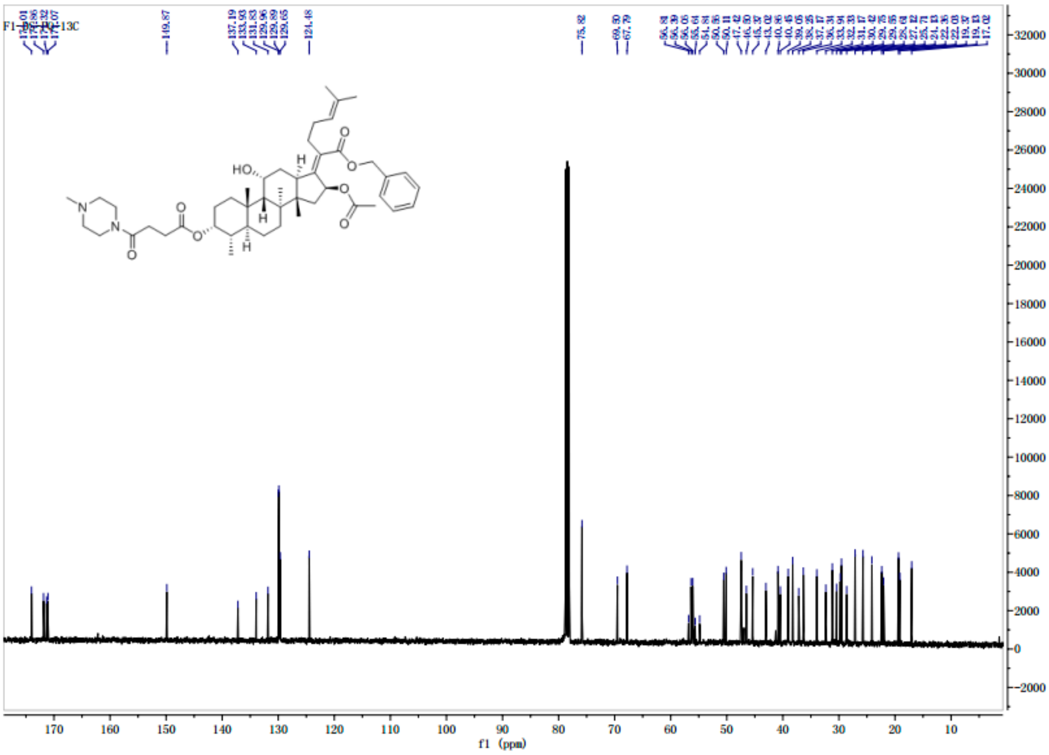
f1 (ppm)

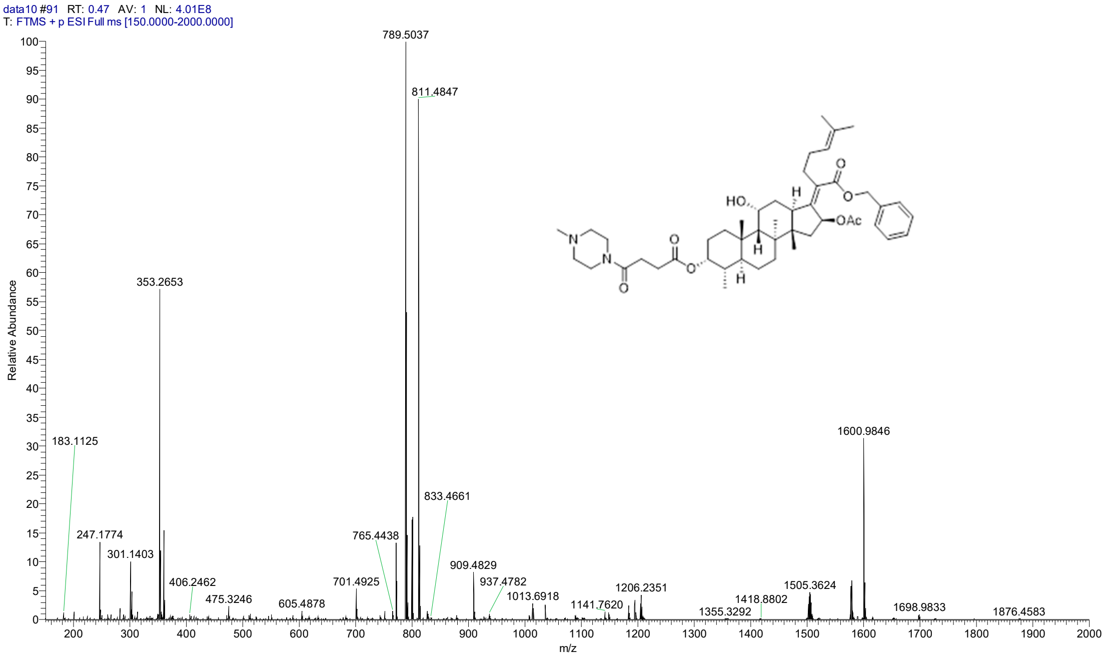

Figure S12. ^{1}H NMR (400 MHz, $CDCl_3$), ^{13}C NMR (100 MHz, $CDCl_3$), and HRMS (ESI) spectra of compound **9.**

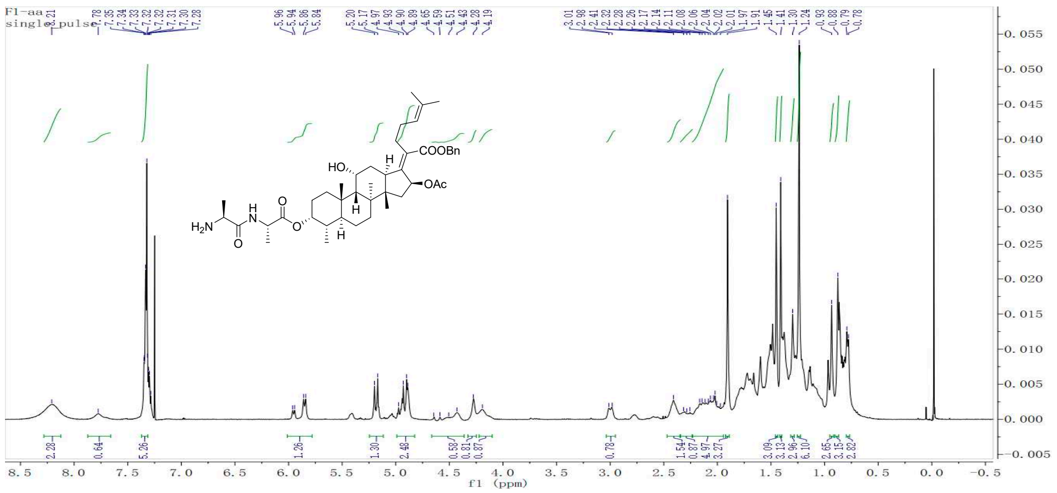

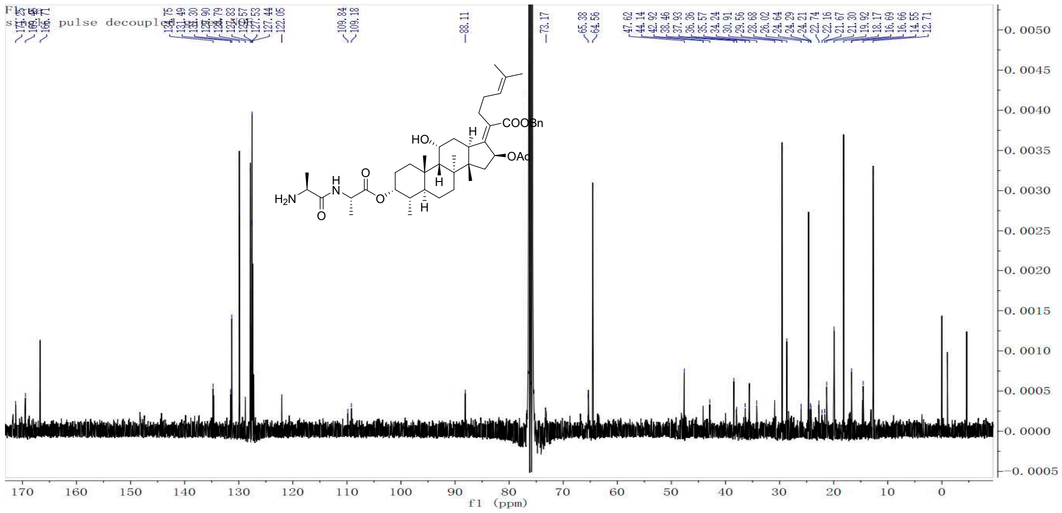

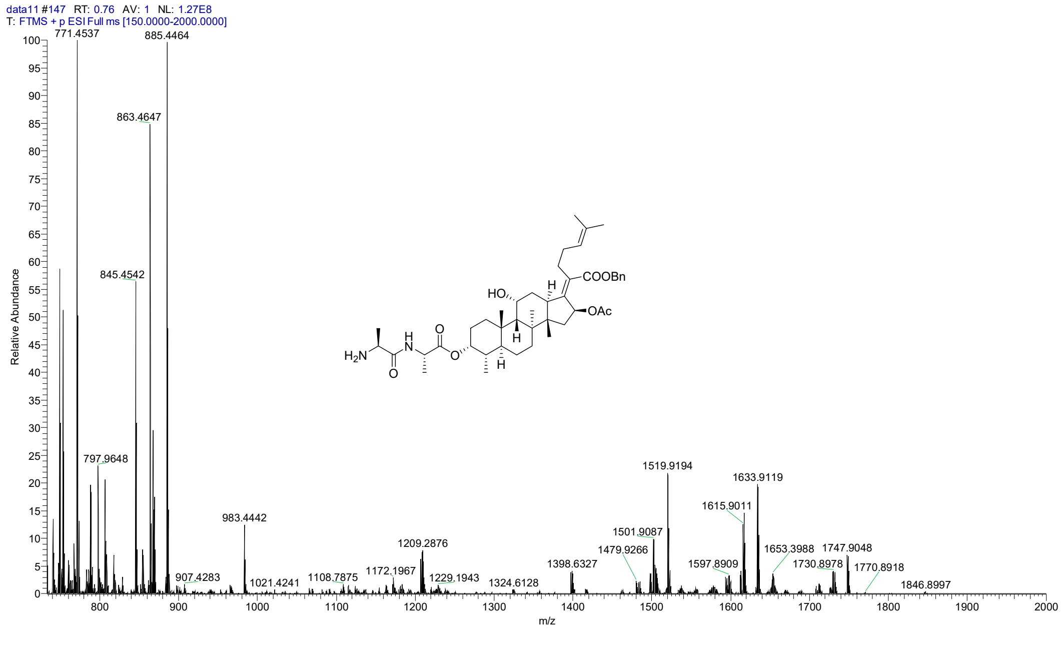

Figure S13. ^{1}H NMR (400 MHz, $CDCl_3$), ^{13}C NMR (100 MHz, $CDCl_3$), and HRMS (ESI) spectra of compound **10.**

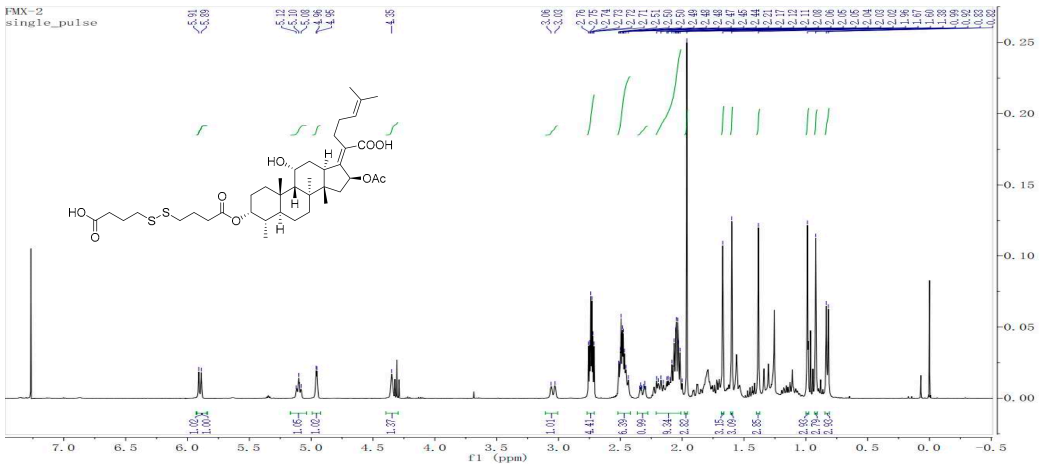

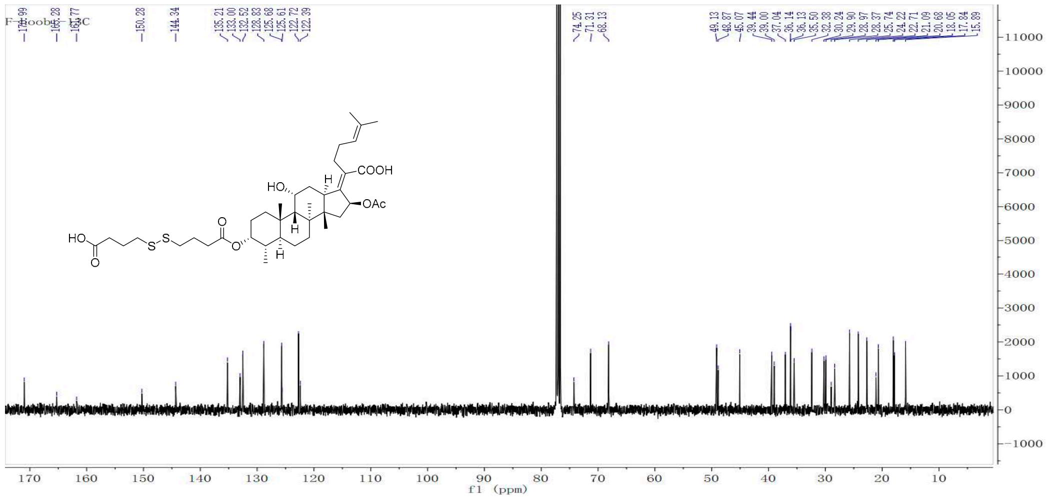

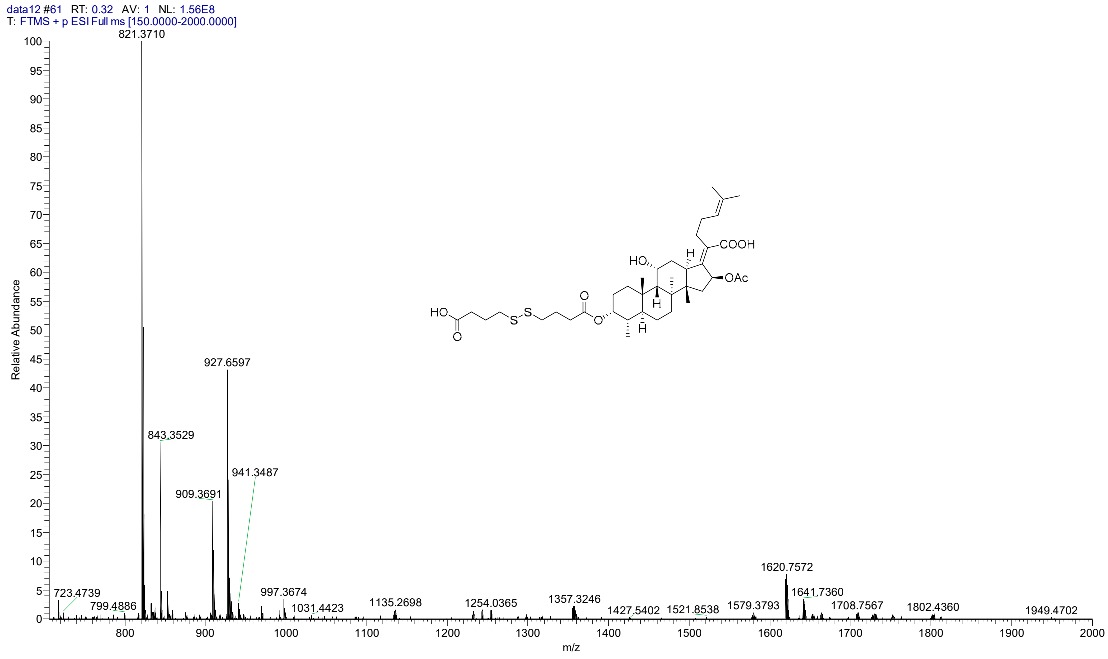

Figure S14. ^{1}H NMR (400 MHz, $CDCl_3$), ^{13}C NMR (100 MHz, $CDCl_3$), and HRMS (ESI) spectra of compound **11.**

Reference

1. Laskowski, R. A.; Swindells, M. B., LigPlot+: multiple ligand-protein interaction diagrams for drug discovery. *Journal of chemical information and modeling* **2011,** *51* (10), 2778-86.
